# TOR Complex 2-Regulated Protein Kinase Fpk1 Stimulates Endocytosis via Inhibition of Ark1/Prk1-Related Protein Kinase Akl1 in Saccharomyces cerevisiae

**DOI:** 10.1128/MCB.00627-16

**Published:** 2017-03-17

**Authors:** Françoise M. Roelants, Kristin L. Leskoske, Ross T. A. Pedersen, Alexander Muir, Jeffrey M.-H. Liu, Gregory C. Finnigan, Jeremy Thorner

**Affiliations:** Divisions of Biochemistry, Biophysics & Structural Biology and Cell & Developmental Biology, Department of Molecular and Cell Biology, University of California, Berkeley, California, USA

**Keywords:** homeostasis, mutants, phosphorylation, plasma membrane, stress response, yeast

## Abstract

Depending on the stress, plasma membrane alterations activate or inhibit yeast target of rapamycin (TOR) complex 2, which, in turn, upregulates or downregulates the activity of its essential downstream effector, protein kinase Ypk1. Through phosphorylation of multiple substrates, Ypk1 controls many processes that restore homeostasis. One such substrate is protein kinase Fpk1, which is negatively regulated by Ypk1. Fpk1 phosphorylates and stimulates flippases that translocate aminoglycerophospholipids from the outer to the inner leaflet of the plasma membrane. Fpk1 has additional roles, but other substrates were uncharacterized. We show that Fpk1 phosphorylates and inhibits protein kinase Akl1, related to protein kinases Ark1 and Prk1, which modulate the dynamics of actin patch-mediated endocytosis. Akl1 has two Fpk1 phosphorylation sites (Ark1 and Prk1 have none) and is hypophosphorylated when Fpk1 is absent. Conversely, under conditions that inactivate TORC2-Ypk1 signaling, which alleviates Fpk1 inhibition, Akl1 is hyperphosphorylated. Monitoring phosphorylation of known Akl1 substrates (Sla1 and Ent2) confirmed that Akl1 is hyperactive when not phosphorylated by Fpk1. Fpk1-mediated negative regulation of Akl1 enhances endocytosis, because an Akl1 mutant immune to Fpk1 phosphorylation causes faster dissociation of Sla1 from actin patches, confers elevated resistance to doxorubicin (a toxic compound whose entry requires endocytosis), and impedes Lucifer yellow uptake (a marker of fluid phase endocytosis). Thus, TORC2-Ypk1, by regulating Fpk1-mediated phosphorylation of Akl1, adjusts the rate of endocytosis.

## INTRODUCTION

Plasma membrane (PM) function requires continual adjustment of its protein and lipid content and distribution. Critical processes, such as exocytosis (1), endocytosis (2) and signaling by ligand-sensing receptors (3), are dramatically impaired if PM constitution is altered. Conversely, the same processes that rely on maintenance of proper PM structure also remodel the PM by eliciting responses that alter the protein and lipid species present (4, 5). Thus, cells must have mechanisms to sense the status of the PM and effector pathways that modulate the reactions needed to maintain homeostasis and preserve the functional state of the PM in the face of changing circumstances and stimuli.

Studies with budding yeast (Saccharomyces cerevisiae) have revealed that the PM-associated target of rapamycin (TOR) complex 2 (TORC2) serves as both a sensor and regulator of PM status. TORC2 activity is stimulated by certain stresses that perturb the PM, including inhibition of sphingolipid synthesis (6–8), hypotonic conditions (7, 9), and heat shock (10), and is inactivated by other PM-perturbing stresses, such as hypertonic conditions (11, 12). Although TORC2 seems to “sense” PM structure, how it does so is unclear. It is thought (7, 9) that the membrane stretch evoked by hypotonic shock activates TORC2 by allowing its association with two ancillary subunits (Slm1 and Slm2) (13–15) normally sequestered within a PM domain, dubbed eisosomes (16), distinct from that harboring TORC2. However, the molecular feature(s) sensed by TORC2 upon this or any other PM perturbation, and the ensuing molecular mechanism by which TORC2 activity is affected in each case, is not fully understood.

Better characterized is how TORC2 controls its sole essential downstream effectors, the protein kinase Ypk1 and its paralog Ypk2/Ykr2 (17–20). Focusing on Ypk1, its basal function requires phosphorylation of T504 in its activation loop by the upstream, eisosome-associated protein kinases Pkh1 and Pkh2 (18, 21–23). Activating PM stresses promote TORC2-mediated phosphorylation of Ypk1 at additional, C-terminal sites, most prominently S644 and T662, which markedly enhance Ypk1 catalytic activity (6, 22); conversely, loss of the modifications installed by TORC2 dramatically dampens Ypk1 activity (11, 12). Strikingly, the lethality arising from the absence of TORC2 activity (24, 25) can be rescued by alleles of Ypk1 (or Ypk2) that bypass the need for their TORC2-mediated phosphorylation (6, 19), indicating that the sole function of TORC2 essential for cell viability is activation of Ypk1 (or Ypk2). Because *YPK1 ypk2*Δ cells are viable and exhibit no overt phenotype, Ypk1 is an effector that can execute all the essential functions of TORC2. Thus, subsequent characterization of the substrates of Ypk1 has shed considerable light on those precincts of cellular physiology that are under the control of the TORC2-Ypk1 signaling axis.

Indeed, elucidation of Ypk1 targets has demonstrated that it is the primary regulator of PM homeostasis. Ypk1 phosphorylates and negatively regulates endocytic adaptors, like Rod1 and Aly2 (8, 26), which promote internalization of nutrient permeases and other classes of integral PM proteins, thereby modulating the protein species in the PM. Ypk1 also phosphorylates and negatively regulates Orm1 and Orm2 (6), which are endoplasmic reticulum (ER)-localized tetraspanins that inhibit the first enzyme unique to sphingolipid biosynthesis (27), thereby increasing flux into this metabolic pathway. In addition, Ypk1 phosphorylates and stimulates ceramide synthase (8), thereby ensuring that pathway upregulation results in efficient production of its complex sphingolipid end products. In addition, Ypk1 phosphorylates and negatively regulates Gpd1 (11), one of two enzymes that convert dihydroxyacetone phosphate into glycerol-3-phosphate (glycerol-3P) (an essential precursor to all glycerophospholipids), as well as phosphorylates and opens the Fps1 glyceroaquaporin that controls glycerol efflux (12), further contributing to control of the supply of this building block for PM glycerophospholipids. Moreover, Ypk1 phosphorylates and negatively regulates Fpk1 and its paralog Fpk2/Kin82 (23), protein kinases responsible, in turn, for phosphorylating and activating Dnf1, Dnf2, and Dnf3 (28), three PM-associated P-type ATPases (flippases) that translocate PM aminoglycerophospholipids from the outer to the inner leaflet (29, 30), thereby influencing the relative content of these lipid species on the two different sides of the PM bilayer. Thus, TORC2-Ypk1 signaling regulates multiple factors that influence the protein and lipid composition, as well as lipid distribution, in the PM.

In addition to its negative regulation by Ypk1-mediated phosphorylation in response to those PM stresses that activate TORC2, Fpk1 is also phosphorylated and negatively regulated by the septin-associated protein kinase Gin4 (31), thereby exerting cell cycle-dependent fine-tuning of the leaflet lipid composition at the bud neck. However, other observations suggest that aside from stimulating flippases, Fpk1 (and Fpk2) act on other substrates. First, a triple mutant lacking Fpk1, Fpk2, and Lem3, an accessory protein required for PM insertion and function of Dnf1 and Dnf2 (29, 30), has a much more severe growth defect than either a *lem3*Δ single mutant or an *fpk1*Δ *fpk2*Δ double mutant (28). Second, Fpk1 phosphorylates Ypk1 at two N-terminal sites in a sphingolipid-dependent manner, but no marked effect on Ypk1 function appears to result from these modifications (23). Third, independent of their action on either Ypk1 or the flippases, Fpk1 and Fpk2 have been implicated in regulation of actin polarization and endocytosis (32); however, no new substrate(s) was identified.

Here we document that Fpk1 phosphorylates and negatively regulates the protein kinase Akl1. Akl1 (1,108 residues) is significantly larger than, but most closely related in its catalytic domain to, the protein kinases Ark1 (638 residues) and Prk1 (810 residues). Ark1 and Prk1 appear to be necessary for uncoating of endocytic vesicles (33), and their orthologs (AAK1 and GAK1) are involved in regulation of clathrin-mediated endocytosis in animal cells (34). We show further that down-modulation of Akl1 by Fpk1 contributes to the efficiency of endocytosis. Our findings define a new physiologically important substrate for Fpk1 and delineate another direct mechanism by which TORC2-Ypk1 signaling (in this case, via its effect on Fpk1 activity) regulates endocytosis, a process intimately coupled to maintenance of PM homeostasis.

## RESULTS

### Identifying and validating Fpk1 substrates.

To date, the only known substrates of Fpk1 (and Fpk2) are (i) the flippases Dnf1, Dnf2, and Dnf3 (hence, the name “flippase protein kinase”) (28) and (ii) the protein kinase Ypk1 (23). We defined the consensus phospho-acceptor site for Fpk1 by determining what residues it phosphorylated in both Ypk1 and Dnf1. This motif is R-X-S-Hpo-D/E, where X represents any amino acid and Hpo represents a hydrophobic residue (L, I, V, M, F, Y, or A) (23), and it is in good accord with subsequent analysis of the phospho-acceptor site preference of Fpk1 (Ynr047w) using synthetic peptide arrays (35). Multiple copies of this motif are found in Dnf1 (six copies), Dnf2 (five copies), and Dnf3 (four copies), many of which have been detected as phosphorylated *in vivo* in genome-wide proteomic analyses (Table 1). We confirmed previously that two such sites (Ser1545 and Ser1552) in the C-terminal cytoplasmic tail of Dnf1 are robustly phosphorylated by Fpk1 *in vitro* (23). However, the physiological importance of phosphorylation at these sites had not been analyzed previously.

**TABLE 1:** S. cerevisiae proteins containing at least two Fpk1 phospho-acceptor site motifs

Protein/ORF	Function	Fpk1 motifs	References describing evidence for phosphorylation *in vivo*
Dnf1/YER166W	Aminoglycerophospholipid translocase (flippase)	RSS^348^LD, RVS^358^AD, RPS^365^LD, RSS^1526^LD, RYS^1545^VE, RTS^1552^LD	23, 68, 98–102
Dnf2/YDR093W	Aminoglycerophospholipid translocase (flippase)	RGS^386^LD, RMS^396^AD, RPS^403^LD, RTS^1566^LD, RAS^1592^LD	23, 32, 68, 98–104
Dnf3/YMR162C	Aminoglycerophospholipid translocase (flippase)	RPS^651^LD, RNS^661^IE, RKS^88^LE, RIS^973^ID	68, 102, 105–107
Ypk1/YKL126W	Ser/Thr protein kinase controlling PM lipid and protein homeostasis	RSS^51^LD, RVS^71^YD	23, 64, 102
Akl1/YBR059C	Ser/Thr protein kinase; member, with Ark1 and Prk1, of a protein kinase family involved in control of endocytosis and actin cytoskeleton organization	RQS^960^LD, RQS^1072^LD	32, 68, 98, 100–104; this study
Cti6/YPL181W	PHD domain-containing component of the Rpd3L histone deacetylase complex	RNS^216^MD, RRS^406^AD	32, 68, 98, 99, 101, 103, 104
Dit2/YDR402C	*N*-Formyltyrosine oxidase	RES^62^ME, RWS^459^LD	
Ent4/YLL038C	Protein of unknown function (contains an N-terminal epsin-like domain)	RQS^182^LE, RFS^213^LD	68, 101, 102, 104
Erg1/YGR175C	Squalene monooxygenase	RPS^302^FD, RKS^386^ID	
Ira2/YOL081W	GTPase-activating protein for Ras1 and Ras2 (paralog of Ira1)	RAS^547^YD, RLS^779^ID	
Irc20/YLR247C	E3 ubiquitin ligase and putative helicase	RKS^152^LE, RFS^224^VE, RES^298^VE	
Lcb5/YLR260w	Long-chain (sphingoid) base kinase (paralog of Lcb4)	RSS^55^ID, RCS^302^IE	68, 98, 102, 104
Mks1/YNL076W	Negative transcriptional regulator (with pleiotropic roles in lysine biosynthetic pathway and nitrogen regulation and in both Ras-cAMP and retrograde [RTG] mitochondrion-to-nucleus signaling)	RLS^453^MD, RQS^518^MD	32, 68, 100–102, 104
Not3/YIL038C	Subunit of CCR4-NOT global transcriptional regulator	RRS^83^VE, RSS^348^AD	
Pfa3/YNL326C	Palmitoyl coenzyme A transferase of the DHHC-CRD family required for C-terminal S-palmitoylation of vacuolar membrane protein Vac8	RPS^322^LE, RAS^329^VE	68, 102
Pkh3/YDR466W	Protein kinase (weak similarity to Pkh1 and Pkh2 and mammalian PDK1)	RIS^283^LE, RNS^499^ID	
Prp2/YNR011C	RNA-dependent DEXD/H-box ATPase required for spliceosome activation	RAS^545^VD, RKS^619^LE	
Tus1/YLR425W	Guanine nucleotide exchange factor for Rho1	RKS^27^IE, RPS^1163^IE	101, 102, 106, 107
Vps54/YDR027C	Component of the Golgi-associated retrograde protein complex	RLS^69^LD, RRS^78^FD	68, 98, 99, 102–104, 108
Yta6/YPL074W	Cortically localized putative AAA^+^ ATPase of the Cdc48/Pas1/Sec18 subfamily	RAS^255^LD, RRS^278^LD, RKS^296^ME	32, 68, 98–101, 104

**FIG 1:**
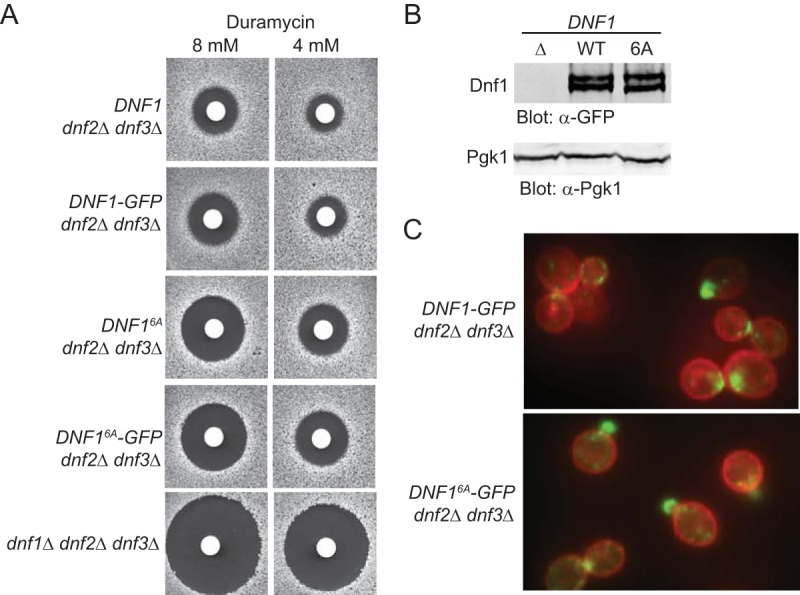
Absence of Fpk1 phosphorylation impairs PtdEth flipping by Dnf1. (A) Strains GFY1770 (*DNF1 dnf2*Δ *dnf3*Δ), GFY1773 (*DNF1-GFP dnf2*Δ *dnf3*Δ), GFY1772 (*DNF1*^*6A*^ *dnf2*Δ *dnf3*Δ), GFY1775 (*DNF1*^*6A*^-*GFP dnf2*Δ *dnf3*Δ), and GFY1728 (*dnf1*Δ *dnf2*Δ *dnf3*Δ) were plated as a lawn on yeast extract-peptone-dextrose (YPD) plates, and 10 μl of stock solution of duramycin (either 8 mM or 4 mM) was spotted onto sterile filter paper disks which were immediately placed onto the lawn. Plates were scanned after incubation at 30°C for 2 days. (B) Extracts from GFY1728, GFY1773, and GFY1775 cells were resolved by SDS-PAGE and analyzed by immunoblotting with anti-GFP antibodies. (C) The same cells as in panel B, costained with CellMask Orange to highlight the plasma membrane, were viewed by fluorescence microscopy as described in Materials and Methods.

The flippases translocate aminoglycerophospholipids from the outer to the inner leaflet of the PM. The more phosphatidylethanolamine (PtdEth) in the outer leaflet, the more sensitive yeast cells are to the killing action of a PtdEth-binding antibiotic, duramycin (36). In the background of a cell in which Dnf2 and Dnf3 are absent, it is clear that Dnf1 makes a major contribution to the inward translocation of PtdEth because loss of Dnf1 makes yeast cells much more sensitive to killing by duramycin (Fig. 1A, compare bottom row to top row). Hence, as one approach to assess whether phosphorylation at its six Fpk1 sites affects Dnf1 function, we used the background of *dnf2*Δ *dnf3*Δ cells to compare the phenotypes of wild-type (WT) Dnf1 and Dnf1-green fluorescent protein (GFP) to those of corresponding site-directed mutants in which the Ser residues in all six Fpk1 motifs had been mutated to Ala. We found that, compared to WT Dnf1 or Dnf1-GFP, the Dnf1(6A) and Dnf1(6A)-GFP mutants were impaired for inward transport of PtdEth, as judged by the readily detectable increase in their sensitivity to duramycin (Fig. 1A). This difference was not attributable to any difference in either the level of expression of Dnf1(6A) compared to that of WT Dnf1 (Fig. 1B) or any difference in the localization pattern of Dnf1(6A)-GFP compared to that of Dnf1-GFP (Fig. 1C). We conclude that phosphorylation at its Fpk1 sites is indeed required for optimal Dnf1 function. [We have evidence that Dnf1 is also a mitogen-activated protein kinase (MAPK) substrate (E. Sartorel, F. M. Roelants, G. C. Finnigan, and J. Thorner, unpublished data); hence, we suspect that the prominent doublet observed, even for Dnf1(6A) lacking all of its Fpk1 sites (Fig. 1B), likely arises from its MAPK-dependent modification.]

Taking advantage of the observations and approaches described above, and cognizant of the indirect evidence that Fpk1 (and Fpk2) may have additional functions (28, 32), we sought to identify previously uncharacterized Fpk1 substrates. To this end, we looked, first, for S. cerevisiae gene products that contain matches to the Fpk1 consensus phospho-acceptor motif using the Pattern Matching tool available at the Saccharomyces Genome Database (http://www.yeastgenome.org/cgi-bin/PATMATCH/nph-patmatch). Second, because Dnf1, Dnf2, Dnf3, and Ypk1 all contain multiple Fpk1 phosphorylation sites, we focused on candidates containing two or more predicted Fpk1 sites. Out of the more than 6,600 apparent open reading frames (ORFs) in the S. cerevisiae genome (http://www.yeastgenome.org/genomesnapshot), only 16 additional proteins contain at least two predicted Fpk1 phosphorylation sites (Table 1).

In particular, one potential candidate, the protein kinase Akl1, drew our attention for several reasons. First, the closest relatives of Akl1 are the protein kinases Ark1 and Prk1 (34), which are involved in regulation of endocytosis and actin cytoskeleton organization (33). Second, Rispal et al. (32) found evidence that Fpk1 and Fpk2 are involved in these same processes, but the mechanism by which they contribute to endocytosis and actin organization was not determined. Third, endocytosis is clearly a process that is intimately connected to PM homeostasis, and we have recently demonstrated that cargo recognition molecules, α-arrestins, required for the endocytosis of integral PM proteins are, like Fpk1, under the direct control of TORC2-Ypk1 signaling (26).

**FIG 2:**
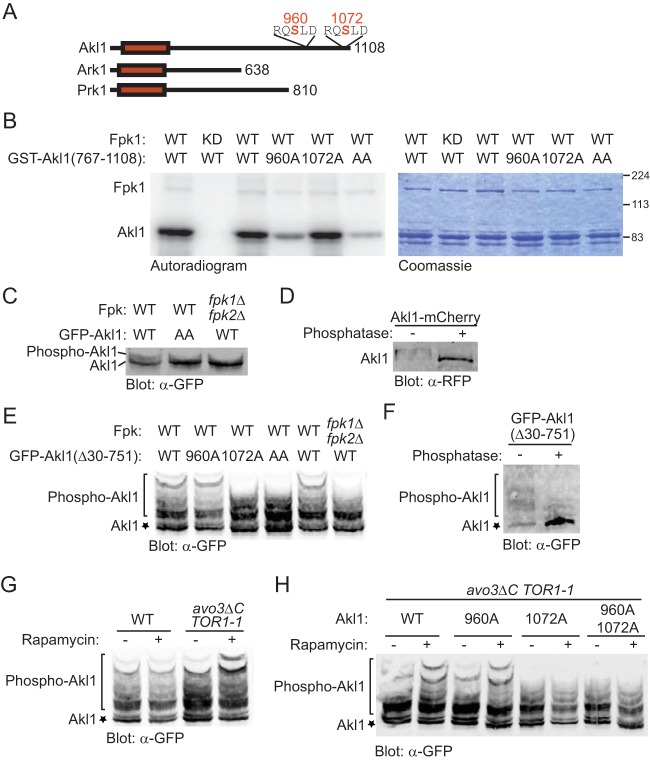
Fpk1 phosphorylates Akl1 at S960 and S1072. (A) Schematic representation of the Akl1, Ark1, and Prk1 protein kinases. The catalytic domain (red rectangle) is near the amino terminus. Akl1 has the longest C-terminal segment, and only Akl1 contains consensus Fpk1 phospho-acceptor site motifs, both near its C terminus. (B) GST-Fpk1 (pAX15, WT) or catalytically inactive (kinase-dead [KD]) mutant GST-Fpk1(D621A) (pJY10) were purified from E. coli, incubated with [γ-^32^P]ATP and either GST-Akl1(767-1108) (pFR290), GST-Akl1(767-1108; S960A) (pFR293), GST-Akl1(767-1108; S1072A) (pFR294), or GST-Akl1(767-1108; S960A S1072A) (pFR297), also purified from E. coli. The resulting products were resolved by SDS-PAGE and analyzed by autoradiography and staining with Coomassie dye. (C) A wild-type (WT) strain (BY4741) and an isogenic *fpk1*Δ *fpk2*Δ mutant (YFR205) expressing from the *GAL1* promoter either GFP-Akl1 (pDD0938) or GFP-Akl1(S960A S1072A) (pFR303) were grown to mid-exponential phase and lysed. The resulting extracts were resolved on a Phos-tag gel and analyzed by immunoblotting with anti-GFP antibodies. (D) Otherwise wild-type cells expressing full-length Akl1-mCherry from its endogenous promoter at its normal chromosomal locus (YFR437) were grown, and extracts were prepared, treated with calf intestinal phosphatase, and then resolved and analyzed as for panel C. (E) Same as in panel C except that the strains were expressing GFP-Akl1 in which residues 30 to 751 were deleted (pFR304) or the same construct with the S960A (pFR329), S1072A (pFR328), or S960A S1072A mutations (pFR334). (F) A wild-type strain (BY4741) expressing GFP-Akl1(Δ30-751) (pFR304) was grown and extracts were prepared, treated with calf intestinal phosphatase, and then resolved and analyzed as for panel E. (G) Strains BY4741 (WT) and *avo3*^Δ*CT*^ *TOR1-1* were transformed with GFP-Akl1(Δ30-751) (pFR304), grown to mid-exponential phase, and left untreated (−) or treated (+) with rapamycin (0.2 μM) for 10 min, which in this strain specifically inhibits TORC2, before being lysed and analyzed as for panel C. (H) *avo3*^Δ*CT*^ *TOR1-1* cells transformed with GFP-Akl1(Δ30-751) (pFR304), GFP-Akl1(Δ30-751; S960A) (pFR329), GFP-Akl1(Δ30-751; S1072A) (pFR328), or GFP-Akl1(Δ30-751; S960A S1072A) (pFR334) were grown and analyzed as for panel G.

Akl1 has an N-terminal catalytic domain (residues 25 to 320) and a long C-terminal extension that contains near the C terminus two canonical matches (RQS^960^LD and RQS^1072^LD) to the consensus Fpk1 phospho-acceptor site motif, whereas Ark1 and Prk1 lack any such sequences (Fig. 2A). Phosphorylation at both sites *in vivo* has been detected in genome-wide proteomic analyses (Table 1). Moreover, the site corresponding to Ser960 in S. cerevisiae Akl1 is highly conserved and the site corresponding to Ser1072 in S. cerevisiae Akl1 is completely conserved in the other sensu stricto Saccharomyces species, as well as in more evolutionarily distant yeasts (see Fig. S1 in the supplemental material, yellow boxes). Furthermore, these sites have been conserved, despite the fact the C-terminal extensions of the Akl1 orthologs of the more distantly related species have clearly diverged substantially from that of S. cerevisiae Akl1, especially compared to the relatively high degree of conservation of their respective kinase domains (Fig. S1). Interestingly, two of the more distantly related yeasts (Saccharomyces castellii and Candida glabrata) have each acquired a third C-terminal consensus Fpk1 site (Fig. S1, underlined in yellow). Despite the very large number of Arg residues in all of these proteins (>50), only those indicated at the immediate C-terminal end, and nowhere else, correspond to an Fpk1 consensus phospho-acceptor site. Hence, we sought to determine whether Akl1 is indeed a bona fide and physiologically relevant substrate of Fpk1.

### Fpk1 phosphorylates Akl1.

We tested first whether Akl1 serves as a substrate for Fpk1 *in vitro*. To avoid the possibility of self-phosphorylation, we generated and purified from Escherichia coli a glutathione *S*-transferase (GST)–Akl1(767-1108) fusion corresponding to the C-terminal 341 residues of Akl1, which contains its consensus Fpk1 sites (but lacks its kinase domain). We found that GST-Akl1(767-1108) incubated with purified recombinant Fpk1 and [γ-^32^P]ATP was robustly phosphorylated but not when incubated with an equivalent amount of a catalytically inactive Fpk1 mutant, Fpk1(D621A), prepared in the same manner (Fig. 2B). The bulk of the phosphorylation occurred at the Fpk1 consensus site corresponding to S960, because mutation of that residue to Ala markedly reduced the amount of radioactivity incorporated, whereas mutation of S1072 to Ala did not (Fig. 2B). However, the corresponding double mutant (S960A S1072A) exhibited a further decrease in incorporation (Fig. 2B), indicating that both sites are phosphorylated *in vitro*.

To assess whether Akl1 is phosphorylated in an Fpk1-dependent manner *in vivo*, we initially analyzed the migration pattern of full-length Akl1 (tagged at its N terminus with GFP) on phosphate affinity (Phos-tag) gels, in which phosphorylated isoforms are retarded in their mobility with respect to the unmodified species (37). Indeed, in cell extracts, we reproducibly detected slower-mobility species, which were largely abrogated in either an Akl1(S960A S1072A) double mutant or an *fpk1*Δ *fpk2*Δ double mutant (Fig. 2C). Slower-mobility species also were observed for full-length Akl1 tagged at its C terminus with mCherry and eliminated by treatment with phosphatase, confirming that they arose from phosphorylation (Fig. 2D). For better resolution of such phospho-isoforms, we next examined the migration of a much smaller (387-residue) derivative, Akl1(Δ30-751). In agreement with the results for full-length Akl1, the slowest-mobility species were eliminated in an *fpk1*Δ *fpk2*Δ double mutant (Fig. 2E, right), as well as by an Akl1(S960A S1072A) double mutation (Fig. 2E, middle). These species result from phosphorylation because they were also eliminated by phosphatase treatment (Fig. 2F). Interestingly, although the GFP-Akl1(Δ30-751) construct examined *in vivo* is largely congruent with and not much larger than the 342-residue GST-Akl1(767-1108) C-terminal fragment used *in vitro*, analysis of the effect of single mutations on the migration pattern indicated that in the cell, S1072 was the primary site for Fpk1-mediated phosphorylation (Fig. 2E, left), whereas S960 was the preferred site *in vitro* (Fig. 2B).

We demonstrated previously that Fpk1 is inhibited by the TORC2-activated protein kinase Ypk1 (23). Therefore, we examined Akl1 phosphorylation in a strain background, *avo3*Δ*CT TOR1-1* (38), in which only TORC2 can be inhibited by treatment of the cells with rapamycin, whereupon Ypk1-mediated inhibition of Fpk1 should be abrogated. Indeed, in accord with our expectations, we found that phosphorylation of Akl1 was increased upon addition of rapamycin in these cells (Fig. 2G). Furthermore, analysis of the S960A, S1072A, and S960A S1072A mutants confirmed that this increase in phosphorylation occurred largely at the S1072 Fpk1 site (Fig. 2H). These findings verify that Fpk1 is activated upon TORC2-Ypk1 inhibition and responsible for the observed increase in phosphorylation.

**FIG 3:**
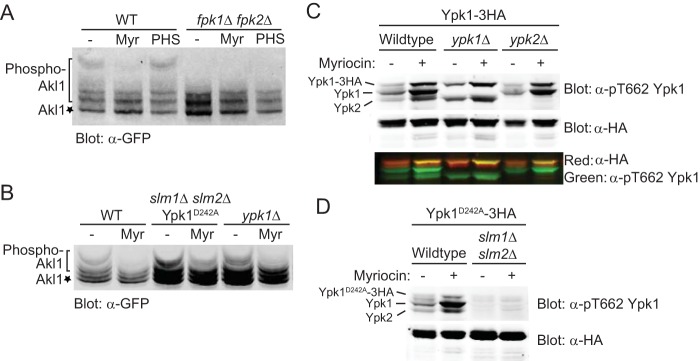
Sphingolipids stimulate Fpk1 function by a mechanism distinct from alleviation of TORC2-Ypk1-mediated inhibition. (A) A wild-type strain (BY4741) and an isogenic *fpk1*Δ *fpk2*Δ mutant (YFR205) expressing from the *GAL1* promoter GFP-Akl1(pDD0938) were grown to mid-exponential phase, and expression was induced with galactose. After 1 h of induction, cells were treated with a vector (−), 1.25 μM myriocin (Myr), or 10 μM phytosphingosine (PHS) for 2 additional hours. The cells were then lysed and analyzed as for Fig. 2E. (B) Same as in panel A, except with WT cells, *slm1*Δ *slm2*Δ cells expressing Ypk1^D242A^ (YFR381), and *ypk1*Δ cells. (C) Anti-Ypk1 phospho-T662 antibodies recognize the TORC2-phosphorylated forms of endogenous Ypk1 and Ypk2 and plasmid-expressed Ypk1-3×HA. Wild-type (BY4741) or otherwise isogenic *ypk1*Δ or *ypk2*Δ cells expressing Ypk1-3×HA (pPL215) were grown to mid-exponential phase and then treated with either a vehicle (methanol) or 1.25 μM myriocin for 2 h to induce TORC2 activation prior to harvesting. Whole-cell extracts were prepared, resolved by Phos-tag SDS-PAGE, and analyzed by immunoblotting with anti-Ypk1 phospho-T662 antibodies and anti-HA.11 epitope antibody. (D) Same as in panel C except that wild-type (BY4741) or *slm1*Δ *slm2*Δ (yKL28) cells expressing Ypk1(D242A)-3×HA (pKL27) were used.

We have provided evidence previously that production of the complex sphingolipid mannosyl-inositolphosphorylceramide (MIPC) is required for maintenance of active Fpk1 *in vivo* (23). Correspondingly, we found that treatment of cells with myriocin (Myr), an antibiotic that inhibits sphingolipid biosynthesis (39), eliminated the slowest-mobility Akl1 phospho-isoforms to an extent similar to that observed in cells lacking Fpk1 and Fpk2, whereas stimulating sphingolipid synthesis by exogenous addition of an excess of the long-chain base phytosphingosine (PHS) modestly elevated the slowest-mobility Akl1 phospho-isoforms and required the presence of Fpk1 and Fpk2 to do so (Fig. 3A). Because blocking sphingolipid production also leads to activation of TORC2, which, in turn, stimulates Ypk1 (6, 7), and Fpk1 is inhibited by Ypk1-mediated phosphorylation (23), it was possible that the apparent decrease in Fpk1 activity observed upon Myr treatment was due solely to upregulation of TORC2-Ypk1-mediated Fpk1 inhibition. However, Myr treatment still caused a marked decrease in the slowest-mobility Akl1 phospho-isoforms in a strain expressing constitutively active Ypk1(D242A) (6) and lacking Slm1 and Slm2 (Fig. 3B) (which prevents TORC2-mediated phosphorylation of Ypk1 or Ypk2 [7, 9]), as we confirmed (Fig. 3C and D). Conversely, Myr treatment still caused a marked decrease in the slowest-mobility Akl1 phospho-isoforms in cells lacking Ypk1 altogether (Fig. 3B). Thus, production of the complex sphingolipid MIPC is needed for optimal Fpk1 activity, independent of the effect that sphingolipids also have on regulation of Fpk1 through TORC2-Ypk1.

Taken together, these collective observations demonstrate unequivocally that Fpk1 phosphorylates Akl1 *in vivo* in a manner responsive to the sphingolipid status of the PM.

### Fpk1-mediated phosphorylation downregulates Akl1 activity.

We next sought to determine the effect that Fpk1-mediated phosphorylation has on Akl1 function. Downstream substrates of Akl1 have been identified and are gene products involved in actin patch-mediated endocytosis (40): Sla1 (41), a protein required for assembly of cortical actin at endocytic sites (33, 42); Pan1 (43, 44), one component of a three-protein complex that stimulates actin filament assembly (45); and Ent1 and Ent2 (46, 47), which are PtdIns4,5P_2_-, ubiquitinylated cargo-, and clathrin-binding proteins (48–50).

**FIG 4:**
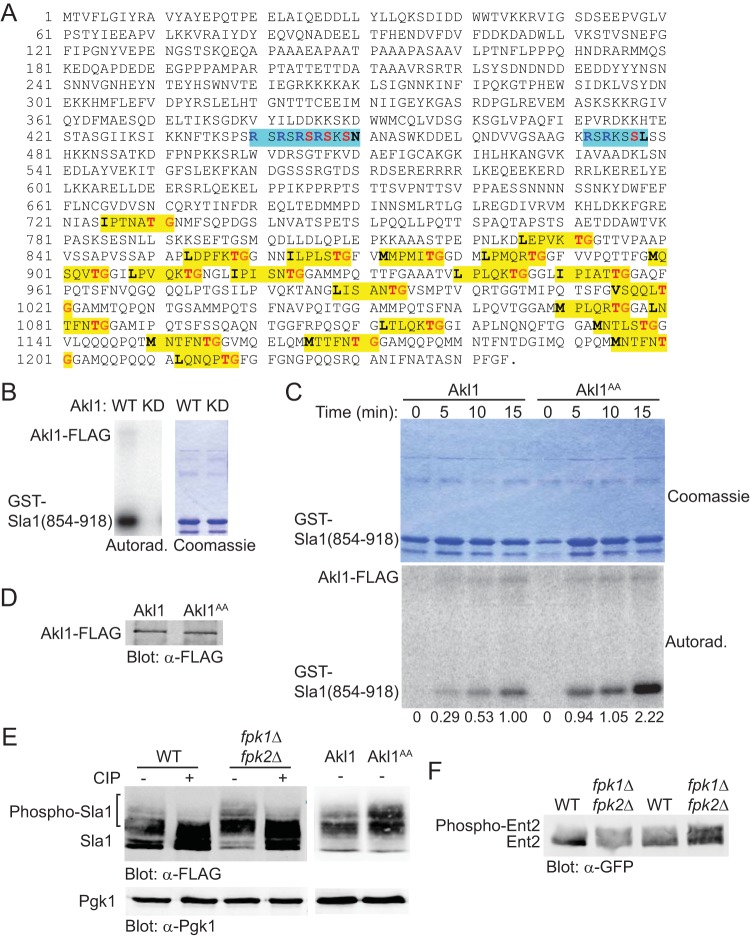
Phosphorylation by Fpk1 downregulates Akl1 activity. (A) Sequence of Sla1. Shown are the consensus Akl1/Prk1 phospho-acceptor site (yellow box), matching either the Prk1 motif (**L/I/V/M**XXQ/N/T/SX**TG**) determined in Pan1 (51) or the Akl1 motif (**L/I/V/M**XXQ/H/M/T/N/AX**TG**) determined using synthetic peptide arrays (35), and the consensus Ypk1 phospho-acceptor sites (turquoise box), **R**X**R**XX**S**[Φ] (where “[Φ]” indicates a preference for a hydrophobic amino acid) (8). (B) Cultures of an *akl1*Δ mutant (YFR479) expressing either Akl1-3×FLAG (pFR316) or a catalytically inactive (kinase-dead) mutant, Akl1(D181Y)-3×FLAG (pFR318), were lysed and the corresponding 3×FLAG-tagged proteins recovered by immunoprecipitation with mouse anti-FLAG antibodies. The resulting immunoprecipitates were incubated with [γ-^32^P]ATP and GST-Sla1(854-918) that had been purified from E. coli harboring plasmid pDD0214. The resulting products were resolved by SDS-PAGE and analyzed as described in Materials and Methods. (C) Same as in panel B except that an *akl1*Δ mutant (YFR479) expressing either Akl1-3×FLAG (pFR316) or Akl1^AA^-3×FLAG (pFR319) was used. Numbers beneath each time point represent the autoradiogram/Coomassie signals, normalized to the ratio observed for WT Akl1 at 15 min. (D) The immunoprecipitates obtained for panel C were resolved by SDS-PAGE and analyzed by immunoblotting with anti-FLAG antibodies. (E) A wild-type (WT) strain (BY4741), an isogenic *fpk1*Δ *fpk2*Δ mutant (YFR205), and strains expressing Akl1 (YFR507) or Akl1^AA^ (YFR508) and expressing from the *GAL1* promoter 3×FLAG-Sla1(851-1244) (pFR360) were grown to mid-exponential phase, and expression was induced with galactose for 3 h. The cells were harvested and lysed, trichloroacetic acid extracts were prepared, and the precipitated proteins were resolubilized, treated with calf intestinal phosphatase (CIP), resolved by SDS-PAGE, and analyzed by immunoblotting. (F) Strains expressing Ent2-GFP (YFR491-A) and Ent2-GFP *fpk1*Δ *fpk2*Δ (YFR492-A) were grown to mid-exponential phase, and cells were harvested and lysed. The resulting extracts were resolved in duplicate on a Phos-tag gel (the rightmost pair of lanes were loaded with 25% more sample than the leftmost pair of lanes) and analyzed by immunoblotting with anti-GFP antibodies.

Sla1 contains 21 consensus Akl1/Prk1 phospho-acceptor site motifs (35, 51), which are all confined to the C-terminal third of this protein (Fig. 4A). We confirmed that a purified recombinant fragment, GST-Sla1(854-918), containing five of these consensus Akl1/Prk1 sites was robustly phosphorylated by WT Akl1-3×FLAG immunoprecipitated from yeast cell extracts but not by a catalytically inactive mutant, Akl1(D181Y)-3×FLAG, prepared in the same manner (Fig. 4B). Hence, as an initial approach to assess the influence of Fpk1-mediated phosphorylation on Akl1 activity, we examined the ability of either WT Akl1-3×FLAG or an equivalent amount of an Akl1(S960A S1072A)-3×FLAG mutant (in which both the Fpk1 phosphorylation sites were mutated to Ala) to phosphorylate GST-Sla1(854-918). A time course for these reactions revealed that Akl1(S960A S1072A)-3×FLAG both autophosphorylated and phosphorylated GST-Sla1(854-918) at a significantly higher rate and to a greater extent than WT Akl1-3×FLAG (Fig. 4C), and immunoblotting confirmed that the mutant and WT kinases were present in the same amount (Fig. 4D). Thus, at least as judged by such *in vitro* reactions, phosphorylation by Fpk1 is inhibitory to Akl1 function.

To confirm this conclusion *in vivo*, and allow for sensitive detection of phospho-isoforms, we examined the mobility of a 394-residue C-terminal fragment of Sla1, 3×FLAG-Sla1(851-1244), which contains 19 of its 21 Akl1 phosphorylation sites (Fig. 4A). We found that in cells lacking Fpk1 and Fpk2, there was an increase in phosphorylation of this fragment, as judged by a readily detectable increase in slower-mobility isoforms and by the marked diminution in the hypophosphorylated isoform, compared to that in the otherwise isogenic *FPK1*^*+*^ *FPK2*^*+*^ cells (Fig. 4E, left). The slower-mobility species were all attributable to phosphorylation because these bands were collapsed by phosphatase treatment (Fig. 4E, left). Likewise, in cells expressing Akl1 in which both of its Fpk1 consensus sites had been mutated to Ala, there was a similar increase in the slower-mobility isoforms compared to the pattern exhibited by otherwise isogenic cells expressing WT Akl1 (Fig. 4E, right). To determine whether this effect was general and applied to other Akl1 substrates, we examined Ent2. Indeed, we saw the same trend; in cells lacking Fpk1 and Fpk2, there was an increase in Ent2 phosphorylation, as judged by an increase in slower-mobility isoforms (Fig. 4F). These data indicate that when Fpk1-mediated phosphorylation is absent, Akl1 is more effective in phosphorylating its substrates in the cell, corroborating the conclusion of our *in vitro* analysis.

**FIG 5:**
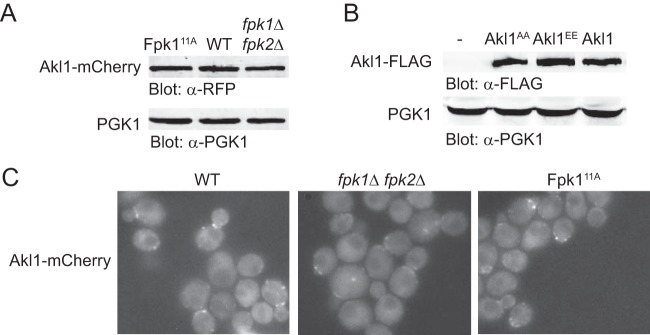
Fpk1 phosphorylation does not affect the stability or localization of Akl1. (A) Extracts from cells expressing Akl1-mCherry (YFR437), Fpk1^11A^ Akl1-mCherry (YFR468) (Fpk1^11A^ is hyperactive because no longer submitted to negative regulation by Gin4 [31]), and *fpk1*Δ *fpk2*Δ Akl1-mCherry (YFR469) were resolved by standard SDS-PAGE and analyzed by immunoblotting with anti-RFP antibodies. (B) Extracts from cells expressing Akl1-3×FLAG (YFR474-A), Fpk1^11A^-3×FLAG (YFR475-A), and Akl1^EE^-3×FLAG (YFR476-A) were resolved by SDS-PAGE and analyzed by immunoblotting with anti-FLAG antibodies. (C) The same strains as in panel A were examined by fluorescence microscopy.

To make certain that these observed effects were mediated by the inhibition that Fpk1-dependent phosphorylation exerts on Akl1 activity *per se*, we examined the impact of Fpk1 function on the level and localization of Akl1. As judged by immunoblotting (and normalized to the loading control, Pgk1), the steady-state level of Akl1 was unaffected, regardless of whether cells expressed a hyperactive Fpk1 allele (Fpk1^11A^) or WT Fpk1 or lacked both Fpk1 and Fpk2 (Fig. 5A). Fpk1^11A^ is hyperactive because it is not subject to negative regulation by Gin4 protein kinase (31). Likewise, when analyzed in the same manner, the levels of an allele lacking its Fpk1 sites, Akl1(S960A S1072A), or an allele mimicking phosphorylation at its Fpk1 sites, Akl1(S960E S1072E), were indistinguishable from those of WT Akl1 (Fig. 5B). Similarly, as judged by fluorescence microscopy, the subcellular distributions of Akl1-mCherry in a number of PM-associated puncta were equivalent in cells expressing WT Fpk1, lacking Fpk1 and Fpk2, or expressing the hyperactive Fpk1^11A^ allele (Fig. 5C). We conclude, therefore, that Fpk1-mediated phosphorylation inhibits Akl1 function but does not affect either its stability or its localization.

### In the absence of negative regulation by Fpk1, Akl1 impedes endocytosis.

**FIG 6:**
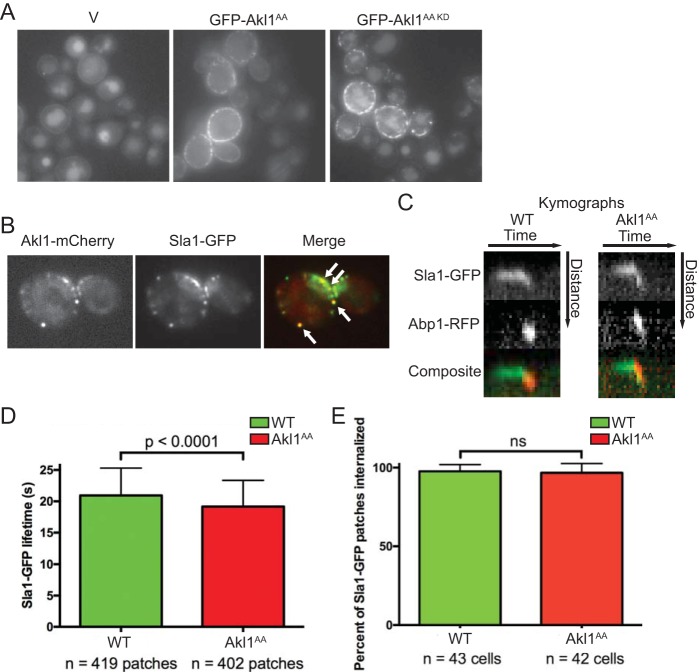
Lack of negative regulation of Akl1 by Fpk1 impedes endocytosis. (A) Cultures of an *akl1*Δ strain (YFR479) containing either an empty vector (V; YCpUG) or expressing from the *GAL1* promoter in the same vector either GFP-Akl1^AA^ (pFR303) or a catalytically inactive (kinase-dead) derivative, GFP-Akl1^AA^(D181A) (pKL31), were induced on galactose medium for 2.5 h, incubated with 4 mg/ml of Lucifer yellow CH (LY) for 30 min at 24°C, and then viewed directly by fluorescence microscopy. (B) Cells (YFR515) expressing both Akl1-mCherry and Sla1-GFP from their endogenous promoters at their normal chromosomal loci were grown on YPD and viewed by fluorescence microscopy. (C) Strains expressing Akl1 Sla1-GFP Abp1-RFP (WT, YFR507) and Akl1^AA^ Sla1-GFP Abp1-RFP (YFR508) were examined by fluorescence video microscopy (see Movies S1 and S2 in the supplemental material), and kymographs were plotted as described in Materials and Methods. (D) The mean Sla1-GFP lifetime at the cell cortex was measured from multiple kymographs as for panel C. (E) The total number of Sla1-GFP patches per cell subsequently joined by Abp1-RFP and moved toward the cell center, a hallmark of actin-driven internalization (40), was determined in cells as in panel C. n, total number cells examined; ns, not significant.

As we have demonstrated here, Sla1 is a substrate for Akl1 both *in vitro* and *in vivo*. Sla1 is essential for proper formation of cortical actin patches (52) and is required for endocytosis (53). Proper execution of endocytosis clearly requires tight spatial and temporal control of a very large number of protein-membrane and protein-protein interactions (40). As for Akl1, all of the known targets of its most closely related protein kinases, Ark1 and Prk1, are involved in clathrin- and actin patch-mediated endocytosis, including Sla1, Pan1, Ent1, Ent2, Yap1801, Yap1802, and Scd5 (41, 46, 51, 54, 55). We confirmed that Akl1-mediated phosphorylation downregulates endocytosis by looking, first, at fluid phase uptake of Lucifer yellow CH (LY) into the vacuole because prior studies established that this dye enters yeast via actin patch-dependent endocytosis (56–58). We found that *akl1*Δ cells carrying an empty *GAL* promoter vector exhibit very prominent LY staining of the vacuole on galactose medium, whereas the same cells expressing Akl1(S960A S1072A) from the *GAL* promoter showed a marked reduction in LY staining of the vacuole, but cells expressing an equivalent level of a catalytically inactive derivative, Akl1(D181A S960A S1072A), did not (Fig. 6A). Moreover, Akl1 localized in cortical “dots” (Fig. 6A), presumably reflecting association with endocytic actin patches, as observed for Ark1 and Prk1 (59). Colocalization of Akl1-mCherry with Sla1-GFP confirmed this conclusion (Fig. 6B). The actions of Ark1 and Prk1 are thought to be involved in recycling of endocytic factors, because in the absence of Ark1 and Prk1, cortical patches containing actin, clathrin, and other endocytic components aggregate at the cell cortex (60) and also accumulate as large clumps in the cytosol (59, 61).

For these reasons, as a test of whether Fpk1 phosphorylation of Akl1 has any effect on endocytosis, we monitored the lifetime of Sla1-GFP at cortical actin patches in cells expressing wild-type Akl1 or the hyperactive Akl1(S960A A1072A) allele, which is immune to Fpk1-mediated inhibition (Fig. 6C; see also Movies S1 and S2). Strikingly, even though these cells contained WT Ark1 and Prk1, there was a modest (11%), yet reproducible and statistically significant (Student's *t* test; *P* = 0.001), reduction in the time that Sla1-GFP remained at cortical actin patches in cells expressing Akl1(S960A A1072A) compared to that in cells expressing WT Akl1 (Fig. 6D), even though the percentage of patches internalized did not vary between cells expressing WT Akl1 and those expressing Akl1(AA) (Fig. 6E). Thus, when not subject to Fpk1-mediated inhibition, Akl1 causes faster dissociation of Sla1 from endocytic sites.

Aside from sequence homology of its kinase domain to that in Ark1 and Prk1, Akl1 was also identified as being encoded by a gene that, when overexpressed, rendered S. cerevisiae resistant to the toxic effects of doxorubicin (43). The following evidence supports the conclusion that this phenotype arises, in large measure, via inhibition of endocytosis. Overexpression of Prk1 also conferred elevated resistance to doxorubicin (43), and it has been shown that elevated Prk1 inhibits endocytosis by dissociating the Sla1/Pan1/End3 complex via phosphorylation of both Sla1 and Pan1 (41, 44). As we have corroborated here, Sla1 is also an efficient substrate for Akl1, and others have shown that Pan1 is a substrate of Akl1 (43). Consistent with this conclusion, Sla1- and End3-defective mutants also exhibited elevated resistance to doxorubicin (43). Conversely, and also consistent with a role for Akl1 in preventing doxorubicin entry by blocking endocytosis, *akl1*Δ cells are hypersensitive to this antibiotic (43, 62, 63).

**FIG 7:**
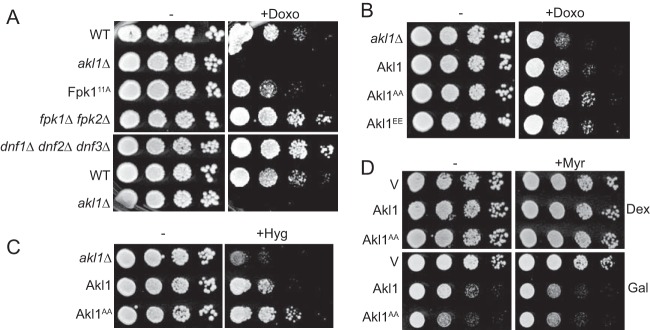
Fpk1 effects on both Akl1 and flippase function influence drug sensitivity. (A) Serial 10-fold dilutions of BY4741 (WT), *akl1*Δ (JTY6532), Fpk1^11A^-expressing (YJW2), *fpk1*Δ *fpk2*Δ (YFR205), and *dnf1*Δ *dnf2*Δ *dnf3*Δ (PFY3272C) cells were spotted on plates lacking (−) or containing (+) doxorubicin (86 μM). The plates were scanned after incubation for 2 days at 30°C. (B) Same as in panel A, except with cells expressing Akl1-3×FLAG (YFR474-A), Akl1^AA^-3×FLAG (YFR475-A), and Akl1^EE^-3×FLAG (YFR476-A) and with a different stock of doxorubicin (which is light sensitive). (C) Serial 10-fold dilutions of *akl1*Δ (YFR479) cells carrying pRS315 (empty vector) or expressing from the same vector Akl1-3×FLAG (pFR316) or Akl1^AA^-3×FLAG (pFR319) were spotted on plates lacking or containing hygromycin B (70 μM). The plates were scanned after incubation for 2 days at 30°C. (D) Serial 10-fold dilutions of *akl1*Δ (YFR479) cells carrying YCpUG (empty vector) or expressing from the *GAL1* promoter GFP-Akl1 (pDD0938) or GFP-Akl1^AA^ (pFR303) were spotted on plates containing dextrose (Dex) or galactose (Gal), lacking or containing 0.8 μM myriocin (+Myr) as indicated. The plates were scanned after incubation for 3 days at 30°C.

Given the role we uncovered for Fpk1 in phosphorylating and negatively regulating Akl1, as a second independent test of whether phosphorylation of Akl1 by Fpk1 has an impact on endocytosis, we examined the effects of doxorubicin on cells with different levels of Fpk1 activity. We found first that cells expressing the hyperactive Fpk1^11A^ allele, which should reduce Akl1 activity and enhance endocytosis, are more sensitive to this compound than otherwise isogenic WT cells (but not as sensitive as an *akl1*Δ null mutant), and conversely, cells lacking Fpk1 and Fpk2, which should permit elevated Akl1 activity and inhibit endocytosis, are more resistant to the compound than WT cells (Fig. 7A, top row). Thus, by this criterion too, Fpk1 modulation of Akl1 activity does influence the efficiency of endocytosis. On the other hand, because Fpk1 also phosphorylates and stimulates the flippases Dnf1 and Dnf2 (28) and because flippase function can affect the permeability of the cell to other xenobiotic agents (31, 64), we tested the effects of doxorubicin on cells lacking the flippases. We found that *dnf1*Δ *dnf2*Δ *dnf3*Δ cells were more doxorubicin resistant than otherwise isogenic WT cells and displayed a degree of resistance quite comparable to that of *fpk1*Δ *fpk2*Δ cells (Fig. 7A, bottom row). Hence, the effects of changes in Fpk1 activity may yield the observed doxorubicin phenotypes through its regulation of Akl1, its regulation of the flippases, or both.

To try to deconvolute these two aspects of Fpk1 function, we compared the doxorubicin sensitivities of *FPK1*^*+*^ *FPK2*^*+*^ cells, which should have normal regulation of flippase function, expressing WT Akl1, an Akl1 allele that cannot be phosphorylated by Fpk1 (which we have shown to be hyperactive compared to WT Akl1), or an Akl1 allele that mimics permanent phosphorylation by Fpk1 (which we presumed would be crippled for function compared to WT Akl1). We found that cells expressing the hyperactive Akl1(S960A S1072A) allele were reproducibly somewhat more resistant to doxorubicin than cells expressing WT Akl1 (Fig. 7B). Thus, in the absence of negative regulation of Akl1 by Fpk1, the increase in phosphorylation of endocytic proteins by Akl1 impedes endocytosis, enhancing doxorubicin resistance. This scenario predicts that an Akl1 mutant that mimics permanent phosphorylation by Fpk1 might be more sensitive to this antibiotic than wild-type cells. However, cells expressing Akl1(S960E S1072E) were just as resistant to doxorubicin as cells expressing Akl1(S960A S1072A). We presume that Glu (or Asp) substitutions are, in this case, not a good mimic for authentic phosphorylation, as we (21, 22) as others (65) have encountered, on occasion, for certain other phosphoproteins.

Cells lacking Akl1 also exhibit greater sensitivity to the killing action of hygromycin B (66). Consistent with our other findings indicating that Akl1(S960A S1072A), which cannot be phosphorylated by Akl1, is hyperactive, we found that Akl1(S960A S1072A) conferred greater resistance to hygromycin B than WT Akl1 (Fig. 7D). Furthermore, prolonged overexpression of Akl1 is toxic to cells (67), and in further accord with the conclusion that Akl1(S960A S1072A) is hyperactive, its overexpression was even more toxic than overexpression of WT Akl1 (Fig. 7D, left side). However, a decrease in sphingolipid levels causes a drastic reduction of Fpk1 activity (23) due to the decrease in the Fpk1 activator MIPC (Fig. 3), as well as to the increase in TORC2-stimulated Ypk1 activity (6) and the ensuing Ypk1-mediated inhibitory phosphorylation of Fpk1 (23). Under these conditions, its Fpk1-mediated negative regulation should be alleviated and, thus, Akl1 maximally active. Consistent with this model, we found that in the presence of the sphingolipid biosynthesis inhibitor myriocin, overexpression of WT Akl1 was just as toxic as overexpression of Akl1(S960A S1072A) (Fig. 7D, right side).

## DISCUSSION

**FIG 8:**
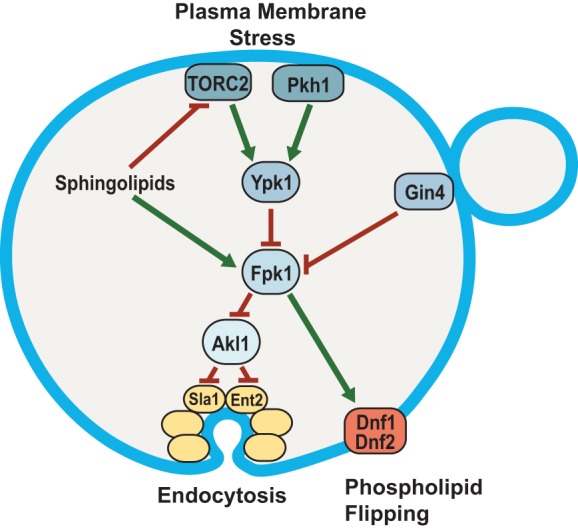
Fpk1 protein kinase is a signaling node that controls membrane permeability and the efficiency of endocytosis. In cells stressed by a diminution in the supply of sphingolipids, Fpk1 is less active for two reasons: (i) lack of direct stimulation by MIPC (23) and (ii) inhibitory phosphorylation by Ypk1, which is activated in a TORC2-dependent manner when sphingolipids are limiting (6, 7). The cell cycle-regulated protein kinase Gin4 also inhibits Fpk1; thus, Fpk1 is also downregulated when Gin4 is active (31). Fpk1, in turn, phosphorylates and stimulates the flippases Dnf1 and Dnf2 and, as shown in this study, phosphorylates and negatively regulates Akl1. One function of Akl1 is to impede endocytosis by phosphorylation of multiple endocytic factors (including Sla1, Ent2, and Pan1), which disables their function. Thus, when Fpk1 activity is high, Akl1 function is downregulated and endocytosis can proceed; when Fpk1 activity is low, Akl1 function is released from inhibition, causing phosphorylation of it targets and thereby down-modulating the efficiency of endocytosis. Two concomitant changes in PM composition ensue when Fpk1 activity is decreased: aminoglycerophospholipid content in the outer leaflet remains higher due to lack of Fpk1-mediated stimulation of the flippases, and bulk membrane and lipid internalization via clathrin-mediated endocytosis is impeded. Both effects contribute to conferring elevated resistance to certain toxic xenobiotic compounds, such as doxorubicin and hygromycin B.

Our findings document that Fpk1 phosphorylates two consensus sites at the C-terminal end of Akl1 *in vitro* and *in vivo* and, further, that this Fpk1-mediated phosphorylation of Akl1 exerts negative regulation that has a readily detectable and physiologically important effect on Akl1 function. When relieved of Fpk1-mediated inhibition, Akl1 action inhibits endocytosis, as judged by more rapid dissociation of an endocytic factor from endocytic sites, elevated doxorubicin resistance, and a reduced rate of LY uptake. Therefore, stimuli that reduce Fpk1 activity, such as sphingolipid depletion and other PM stresses that activate TORC2-Ypk1 action, or cell cycle-dependent activation of protein kinase Gin4, which also phosphorylates and negatively regulates Fpk1 (31), down-modulate the efficiency of endocytosis (Fig. 8). This response perhaps makes biological sense, in that cells would avoid drastic removal of PM constituents while undergoing other processes to cope with the stress.

Until now, Akl1 was the least studied of the three Ark1 and Prk1 family protein kinases, mainly because lack of both Ark1 and Prk1 causes a prominent endocytic defect and major actin assembly aberrations, which are not observed when Akl1 is deleted, either alone or in combination with either an *ark1*Δ or *prk1*Δ mutation (59, 61). Thus, under otherwise normal growth conditions, Akl1 makes, at most, only a minor contribution to modulating actin patch-mediated endocytosis. However, our results reveal that the role of Akl1 is to provide a mechanism whereby cells can down-modulate the efficiency of endocytosis in response to membrane stress. The factor that couples Akl1 to these stress response pathways is the protein kinase Fpk1, whose activity is, in turn, a nodal point for inputs from several upstream signaling pathways (Fig. 8).

A recent study (32) also linked TORC2 activity to the phosphorylation state of Akl1, but that study reported, contrary to our findings, that Akl1 was less phosphorylated when TORC2 was inhibited. In their global mass spectrometry analysis, however, the only residue hypophosphorylated upon TORC2 inhibition was the S in the motif -EQS^504^PR-, which is likely a Cdk1 site, because it has been shown to be modified in a *CDC28*-dependent manner *in vivo* (68) and is not well conserved among Akl1 orthologs (Fig. S1). Apparently, Rispal et al. did not detect phosphorylation sites in Akl1 that increased.

Given the regulatory circuit that we have described here (Fig. 8), inhibition of TORC2 should reduce phosphorylation of authentic Akl1 substrates. However, any such changes are likely masked by the fact that Ark1 and especially Prk1 share the same consensus phospho-acceptor motif and are not under the same regulation. In agreement with this view, in the mass spectrometry analysis conducted by Rispal et al. (32), Sla1, a protein that we demonstrated here is a bona fide Akl1 substrate both *in vitro* and *in vivo*, did not show any diminution of phosphorylation at its Akl1/Prk1 consensus sites after TORC2 inhibition. Interestingly, they did observe hypophosphorylation within peptide sequences in Sla1 that contain its consensus Ypk1 phospho-acceptor site motifs (Fig. 4A), suggesting that Sla1 might also be a direct target of Ypk1. However, Rispal et al. were unable to show direct phosphorylation of Sla1 by Ypk1 *in vitro* (32) and overexpression of Sla1 is not growth inhibitory when Ypk1 activity is limiting, a hallmark of many demonstrated Ypk1 substrates (8). Nonetheless, in a recent global proteomics study (69), myriocin treatment, which activates TORC2 and Ypk1 (6), increased Sla1 phosphorylation at S449, a candidate Ypk1 site (Fig. 4A).

Most strikingly, we have shown here that the C-terminal 394 residues of Sla1, which contain 19 of its 21 Akl1/Prk1 consensus sites (but no Ypk1 consensus site [Fig. 4A]), are clearly hyperphosphorylated when Akl1 cannot be phosphorylated by Fpk1 (Fig. 4E). Thus, as we have shown for Sla1 (Fig. 6C and D), stress-induced activation of the TORC2→Ypk1—|Fpk1—|Akl1 kinase circuit (the arrow indicates a positive [activating] effect, and the T-bars indicate a negative [inhibitory] effect) will increase phosphorylation of other endocytic factors by Akl1, causing their premature dissociation and thereby impeding clathrin- and actin patch-mediated endocytosis. In agreement with this conclusion, it has been shown recently (70) that a Sla1 derivative lacking 10 of its 21 C-terminal Akl1/Prk1 phosphorylation sites has a substantially longer dwell time on cortical actin patches than WT Sla1.

Several other candidate Fpk1 substrates (Table 1) have described roles in processes that could affect the efficiency of endocytosis indirectly because adequate sphingolipid and sterol production are both required for normal endocytic events (71, 72). For example, Vps54 is a component of the GARP complex, which has recently been shown to be required for sphingolipid homeostasis (73); Lcb5 is a long-chain (sphingoid) base kinase (74), and Erg1 is the squalene monooxygenase that generates the squalene epoxide required for ergosterol biosynthesis (75). Further study of such putative Fpk1 substrates could deepen our understanding of how the TORC2-Ypk1-Fpk1 signaling circuit controls PM homeostasis.

We found that hyperactive Akl1 enhances resistance to doxorubicin, a widely used cytotoxic anticancer medicine. As with many chemotherapeutic agents, acquisition of resistance is a major problem. Doxorubicin resistance in human cells is due, in some cases, to upregulation of ABC transporters (multidrug resistance pumps) MRP1 (76) and MDR1/P-glycoprotein (77), but other proteins also have been implicated (78, 79). A prior study with S. cerevisiae was the first to show that overexpression of either Akl1 or Prk1, and consequent inhibition of endocytosis, conferred enhanced resistance to doxorubicin (43). The same study reported that overexpression of AAK1, a human Ark1/Prk1-related family member, conferred elevated doxorubicin resistance in human cells (43). Thus, in a therapeutic setting, use of kinase inhibitors that inhibit AAK1 and GAK1 activity might enhance the effectiveness of doxorubicin as an antitumor agent. In the same regard, as we have shown here (Fig. 3), maintenance of high Fpk1 activity (and, thus, inhibition of Akl1) requires robust sphingolipid biogenesis and, in accordance, cells lacking Orm2, which increases metabolic flux into sphingolipid biosynthesis (27, 80), are more sensitive to doxorubicin than wild-type cells (81).

We also showed here that deletion of Dnf1, Dnf2, and Dnf3 renders cells highly resistant to doxorubicin (Fig. 7A), highlighting the importance of flippase action and thus the outer and inner leaflet aminoglycerophospholipid composition in sensitivity to this drug. It was suggested that flippases have a direct role in endocytosis (82), but it seems more likely that flippase action modulates the leaflet lipid composition, thereby influencing the efficiency with which xenobiotic agents partition into and cross the PM permeability barrier. The bilayer lipid distribution likely also influences membrane protein composition, distribution, and/or orientation, with consequent effects on the content and function of many integral PM-localized proteins, like drug efflux pumps. Pdr5, the ABC transporter most responsible for pleiotropic drug resistance in S. cerevisiae, has been implicated in doxorubicin resistance (63, 83–85), but direct interactions between flippases and Pdr5 (or other PM-localized ABC transporters) have been reported (86–88). Therefore, the resistance to doxorubicin of cells overexpressing Pdr5 (89) may be due not solely to increased Pdr5-mediated ejection of the drug but possibly also to negative effects on flippase activity caused by elevated Pdr5.

## MATERIALS AND METHODS

### Strains and growth conditions.

Yeast strains used in this study (Table 2) were grown routinely at 30°C unless otherwise indicated. Yeasts were cultivated on standard rich (yeast extract-peptone [YP]) medium or on a defined minimal (synthetic complete [SC]) medium (90) supplemented with appropriate nutrients to maintain selection for plasmids, using 2% glucose (Glc) as the carbon source unless otherwise indicated. For gene induction from the *GAL1* promoter, cells were pregrown to mid-exponential phase in SC medium containing 2% raffinose and 0.2% sucrose, galactose (Gal) was added (2% final concentration), and incubation was continued for 3 h. When cells were treated with myriocin (Myr; Sigma-Aldrich Co., St. Louis, MO) or phytosphingosine (PHS; Avanti Polar Lipids, Inc., Alabaster, AL), the cultures were grown to mid-exponential phase and induced with Gal for 1 h, the compounds added at the final concentrations indicated (Myr, 1.25 μM, and PHS, 10 μM), and incubation was continued for a further 2 h. Standard yeast genetic methods were used for strain construction (90).

**TABLE 2:** S. cerevisiae strains used in this study

Strain	Genotype	Source or reference
BY4741	*MAT***a** *his3*Δ*1 leu2*Δ*0 met15*Δ*0 ura3*Δ*0*	Research Genetics, Inc.
YFR205	BY4741 *fpk1*Δ::KanMX4 *fpk2*Δ::KanMX4 *lys2*Δ*0*	23
YJW2	BY4741 Fpk1^11A^::*HIS3*	31
YFR437	BY4741 Akl1-mCherry::*CaURA3*	This study
YFR468	BY4741 Fpk1^11A^::*HIS3* Akl1-mCherry::*CaURA3*	This study
YFR469	BY4741 *fpk1*Δ::KanMX4 *fpk2*Δ::KanMX4 Akl1-mCherry::*CaURA3*	This study
YFR515	BY4741 Akl1-mCherry::*CaURA3* Sla1-GFP::*HIS3*	This study
PFY3272C	BY4741 *dnf1*Δ::KanMX4 *dnf2*Δ::KanMX4 *dnf3*Δ::KanMX4	109
*ypk1*Δ mutant	BY4741 *ypk1*Δ::KanMX4	Research Genetics, Inc.
yKL28	BY4741 *slm1*Δ::KanMX *slm2*Δ::KanMX [pRS416-Ypk1(D242A)-3HA]	This study
GFY1770	BY4741 *DNF1*::HygMX *dnf2*Δ::KanMX *dnf3*Δ::KanMX	This study
GFY1773	BY4741 *DNF1-GFP*::HygMX *dnf2*Δ::KanMX *dnf3*Δ::KanMX	This study
GFY1772	BY4741 *DNF1*(*S348A S358A S365A S1526A S1545A S1552A*)::HygMX *dnf2*Δ::KanMX *dnf3*Δ::KanMX	This study
GFY1775	BY4741 *DNF1*(*S348A S358A S365A S1526A S1545A S1552A*)-*GFP*::HygMX *dnf2*Δ::KanMX *dnf3*Δ::KanMX	This study
GFY1728	BY4741 *dnf1*Δ::natNT2 *dnf2*Δ::KanMX *dnf3*Δ::KanMX	This study
BY4742	*MAT*α *his3*Δ*1 leu2*Δ*0 lys2*Δ*0 ura3*Δ*0*	Research Genetics, Inc.
*ypk2*Δ mutant	BY4742 *ypk2*Δ::KanMX4	Research Genetics, Inc.
YFR381	BY4742 *slm1*Δ::KanMX4 *slm2*Δ::KanMX4 [pRS315-Ypk1(D242A)-Myc]	This study
JTY6532	BY4742 *akl1*Δ::KanMX	Research Genetics, Inc.
YFR474-A	BY4742 Akl1-3×FLAG::*URA3*	This study
YFR475-A	BY4742 Akl1(S960A S1072A)-3×FLAG::*URA3*	This study
YFR476-A	BY4742 Akl1(S960E S1072E)-3×FLAG::*URA3*	This study
YFR479	BY4742 *akl1*Δ::Hyg^r^	This study
*avo3*ΔCT *TOR1-1* mutant	*MAT***a** *avo3*Δ1274-1430 *TOR1-1 trp1 his3 ura3 leu2 rme1* (TB50 strain background)	38
YFR491-A	*MAT***a** *ENT2-GFP*::*HIS3 his3 leu2 ura3 met15*	This study
YFR492-A	*MAT***a** *ENT2-GFP*::*HIS3 fpk1*Δ::KanMX4 *fpk2*Δ::KanMX4 *his3 leu2 ura3 lys2*	This study
JTY5180	*MAT*α *ABP1-RFP*::*HIS3 his3*-Δ*200 ura3-52 leu2-3,112* (DDY3058)	110
YFR507	*MAT***a** Akl1::*URA3 Sla1-GFP*::*CgHIS Abp1-RFP*::*HIS3 his3*-Δ*200 ura3-52 leu2-3,112 lys2-801*	This study
YFR508	*MAT***a** Akl1(S960A S1072A)::*URA3 Sla1-GFP*::*CgHIS Abp1-RFP*::*HIS3 his3*-Δ*200 ura3-52 leu2-3,112 lys2-801*	This study

### Plasmids and recombinant DNA methods.

Plasmids used in this study (Table 3) were constructed using standard procedures (91) in E. coli strain DH5α. The fidelity of all constructs was verified by nucleotide sequence analysis. All PCRs were performed using Phusion DNA polymerase (Thermo Fisher Scientific, Inc., Waltham, MA) or high-fidelity KOD Hot Start DNA polymerase (EMD Millipore, Billerica, MA). Site-directed mutagenesis using synthetic mismatch oligonucleotide primers, as appropriate, was conducted using the QuikChange method (Agilent Technologies, Inc., Santa Clara, CA).

**TABLE 3:** Plasmids used in this study

Plasmid	Description	Source or reference
pGEX4T-1	GST tag, bacterial expression vector	GE Healthcare, Inc.
pAX15	pGEX4T-1 Fpk1-GSGSHHHHHH	This study
pJY10	pGEX4T-1 Fpk1(D621A)-GSGSHHHHHH	This study
pFR290	pGEX4T-1 Akl1(767-1108)	This study
pFR293	pGEX4T-1 Akl1(767-1108; S960A)	This study
pFR294	pGEX4T-1 Akl1(767-1108; S1072A)	This study
pFR297	pGEX4T-1 Akl1(767-1108; S960A S1072A)	This study
pGEX3	GST tag, bacterial expression vector	GE Healthcare, Inc.
pDD0214	pGEX3 Sla1(854-918)	Drubin lab, UC Berkeley
pTS408	*CEN URA3 GAL1_prom_* GFP vector	111
pDD0938	pTS408 GFP-Akl1	Drubin lab, UC Berkeley
pFR303	pTS408 GFP-Akl1(S960A S1072A)	This study
pKL31	pTS408 GFP-Akl1(D181A S960A S1072A)	This study
pFR304	pTS408 GFP-Akl1(Δ30-751)	This study
pFR329	pTS408 GFP-Akl1(Δ30-751; S960A)	This study
pFR328	pTS408 GFP-Akl1(Δ30-751; S1072A)	This study
pFR334	pTS408 GFP-Akl1(Δ30-751; S960A S1072A)	This study
pRS315	*CEN LEU2*	112
pFR234	pRS315 Ypk1(D242A)-Myc	This study
pFR316	pRS315 Akl1-3×FLAG	This study
pFR318	pRS315 Akl1(D181Y)-3×FLAG	This study
pFR319	pRS315 Akl1(S960A S1072A)-3×FLAG	This study
YCpLG	*CEN LEU2 GAL*1*_prom_* vector	113
pFR360	YCpLG 3×FLAG-Sla1(851-1244)	This study
pRS416	*CEN URA3* vector	112
pPL215	pRS416 *MET25_prom_* Ypk1-3×HA	9
pKL27	pRS416 *MET25_prom_* Ypk1(D242A)-3×HA	This study

### Preparation of cell extracts and immunoblotting.

The cells in samples (1.5 ml) of an exponentially growing culture (*A*_600_ = 0.6) were collected by brief centrifugation, immediately frozen in liquid N_2_, and then lysed by resuspension in 150 μl of 1.85 M NaOH and 7.4% β-mercaptoethanol. Protein in the resulting lysate was precipitated by the addition of 150 μl of 50% trichloroacetic acid on ice. After 10 min, the resulting denatured protein was collected by centrifugation, washed twice with acetone, and solubilized by resuspension in 80 μl of 5% SDS in 0.1 M Tris base, and then 20 μl of a 5× stock of SDS-PAGE sample buffer was added. After boiling for 5 min, portions (15 μl) of the samples of interest were resolved electrophoretically, as follows: GFP-Akl1 or GFP-Akl1(Δ30-751) on a Phos-tag gel (8% acrylamide, 29:1 monomer–cross-linker, 23 μM Phos-tag reagent [Wako Pure Chemical Industries, Ltd., Osaka, Japan]); Akl1-mCherry, Akl1-3×FLAG, or Ypk1-3×HA by SDS-PAGE (8% acrylamide, 29:1 monomer–cross-linker); 3×FLAG-Sla1(851-1244) by SDS-PAGE (10% acrylamide, 79:1 monomer–cross-linker); and Ent2-GFP on a Phos-tag gel (8% acrylamide, 29:1 monomer–cross-linker, 10 μM Phos-tag reagent). For immunoblotting, the resolved proteins were transferred to nitrocellulose paper and the resulting filters incubated with appropriate primary antibodies in Odyssey buffer (Li-Cor Biosciences, Inc., Lincoln, NE), washed, incubated with appropriate secondary antibodies conjugated to infrared fluorophores, and visualized using an Odyssey infrared imaging system (Li-Cor Biosciences). Antibodies used (dilution indicated) were mouse anti-FLAG monoclonal antibody (MAb) M2 (1:10,000; Sigma-Aldrich), mouse anti-GFP MAb (1:1,000; Roche Diagnostics, Inc., Indianapolis, IN), anti-red fluorescent protein (anti-RFP; 1:10,000; Rockland Immunochemicals, Inc., Boyertown, PA), rabbit polyclonal anti-Ypk1 phospho-T662 antibodies (9) (1:20,000; gift of Ted Powers, University of California, Davis), mouse monoclonal anti-HA.11 epitope antibody (1:10,000; BioLegend, Inc., San Diego, CA), and rabbit anti-Pgk1 (1:10,000; this lab, prepared as described in reference 92).

### Expression and purification of GST-Fpk1-6×His.

GST-Fpk1-6×His or a kinase-dead derivative, GST-Fpk1(D621A)-6×His, was expressed from vector pGEX4T-1 (GE Healthcare Life Sciences, Chicago, IL) in E. coli strain BL21(DE3). Bacterial cultures (1 liter) in Luria-Bertani (LB) broth supplemented with ampicillin to select for the expression plasmid were grown to mid-exponential phase (*A*_600_ = ∼0.7), and protein production was induced by addition of 1.2 mM isopropyl-β-d-thiogalactoside. After incubation with vigorous aeration for 6 h at 30°C in a controlled-temperature room, the cells were collected by centrifugation and washed with 30 ml of ice-cold lysis buffer (phosphate-buffered saline [PBS; pH 7.4], 2 mM MgCl_2_, 1 mM EDTA, 0.5% [vol/vol] Tween 20, 1× cOmplete protease inhibitors [Roche]) and recollected by centrifugation. The cell pellet was then frozen in liquid N_2_ and stored at −80°C. Cells were subsequently thawed in 50 ml of ice-cold lysis buffer and ruptured at 4°C by sonication using an acoustic cell disrupter (model W185D; Branson Ultrasonics Corp., Danbury, CT). The resulting lysate was clarified by centrifugation at 13,000 × *g* for 30 min at 4**°**C. The clarified extract was then incubated with 2 ml of a slurry of glutathione-agarose beads (GE Healthcare Life Sciences) in PBS (1:1) for 1.5 h at 4°C. The mixture was then decanted into a glass column (1.5-cm diameter; Bio-Rad Laboratories, Inc., Hercules, CA). After removal of the flowthrough, the resin bed was rinsed with 10 ml of wash buffer (phosphate-buffered saline [pH 7.4], 1 mM dithiothreitol [DTT], 0.1% [vol/vol] Tween 20) and the bound protein was eluted by addition of 3 ml of wash buffer containing 30 mM freshly dissolved glutathione. The protein eluate was diluted with 6 ml of HisA buffer (phosphate-buffered saline [pH 7.4], 0.1% [vol/vol] Tween 20, 20 mM imidazole) and loaded using the 10-ml loop onto a 1-ml HisTRAP HP column in an AKTA fast-performance liquid chromatography (FPLC) system (GE Healthcare Life Sciences). The column was eluted with a linear gradient of HisA buffer to HisB buffer (phosphate-buffered saline [pH 7.4], 0.1% [vol/vol] Tween 20, 500 mM imidazole), and fractions were collected. The proteins present in each fraction were resolved by SDS-PAGE and visualized by staining with Coomassie blue dye. The peak fractions containing the highest concentrations of GST-Fpk1-6×His [or GST-Fpk1(D621A)-6×His] were pooled, and to exchange the buffer, the purified protein was passed over a PD-10 desalting column (GE Healthcare Life Sciences) preequilibrated in storage buffer (50 mM Tris-Cl [pH 7.5], 150 mM NaCl, 20% glycerol). Aliquots of the purified protein solution were frozen in liquid N_2_ and stored at −80°C. Protein concentration was determined by the Bradford assay method (Bio-Rad), and the degree of purity was assessed by SDS-PAGE followed by Coomassie staining.

### Expression and purification of GST-Akl1(767-1108) and GST-Sla1(854-918).

Freshly transformed E. coli BL21(DE3) cells carrying a plasmid expressing the desired GST fusion protein were grown to an *A*_600_ of 0.6, and protein production was induced by addition of isopropyl-β-d-thiogalactopyranoside (0.6 mM final concentration). After vigorous aeration for 4 h at 30°C, cells were harvested and the GST fusion protein was purified by column chromatography on glutathione-agarose beads using the procedure described in the preceding section.

### Protein kinase assay in solution.

Purified GST-Fpk1-6×His, or the kinase-dead derivative, GST-Fpk1(D621A)-6×His, was incubated at 30°C in protein kinase assay buffer (125 mM potassium acetate, 12 mM MgCl_2_, 0.5 mM EDTA, 0.5 mM EGTA, 2 mM DTT, 1% glycerol, 0.02% bovine serum albumin [BSA], 25 mM β-glycerol phosphate, 1 mM sodium orthovanadate, 20 mM Tris-HCl [final pH 7.2]) with 100 μM [γ-^32^P]ATP (∼5 × 10^5^ cpm/nmol) and 0.5 μg of purified GST-Akl1(767-1108). After 30 min, reactions were terminated by addition of SDS-PAGE sample buffer containing 6% SDS, followed by boiling for 5 min. Labeled proteins were resolved by SDS-PAGE and analyzed by autoradiography using a PhosphorImager (Molecular Dynamics Division, Amersham Pharmacia Biotech, Inc., Piscataway, NJ).

### Immune complex protein kinase assay.

Cultures (40 ml) of yeast strain *akl1*Δ (YFR479) expressing either Akl1-3×FLAG (pFR316) or Akl1^AA^-3×FLAG (pFR319) were grown to mid-exponential phase, collected by centrifugation, washed in ice-cold 1× PBS, resuspended in 0.2 ml of ice-cold immunoprecipitation (IP) buffer (20 mM Tris-HCl [pH 7.5], 125 mM potassium acetate, 0.5 mM EDTA, 0.5 mM EGTA, 1 mM DTT, 0.1% Tween 20) containing protease inhibitors (complete EDTA free; Roche) and phosphatase inhibitors (25 mM β-glycerol phosphate and 1 mM sodium orthovanadate), lysed, and immunoprecipitated with M2 anti-FLAG immunoglobulin-coated protein A/G beads (Calbiochem-Novabiochem International, Inc., San Diego, CA) as described previously (18). Bead-bound immune complexes were collected by centrifugation, washed once with IP buffer and twice with the kinase assay buffer described in the preceding section, resuspended in 20 μl of kinase assay buffer containing 100 μM [γ-^32^P]ATP (∼5 × 10^5^ cpm/nmol), and incubated for 30 min with 0.5 μg of purified GST-Sla1(854-918). Reactions were terminated by addition of SDS-PAGE sample buffer containing 6% SDS, followed by boiling for 5 min. Labeled proteins were resolved and analyzed by autoradiography as described in the preceding section.

### Subcellular localization by fluorescence microscopy.

Yeast proteins fused to Aequoria victoria green fluorescent protein (GFP) (93) or Discosoma sp. red fluorescent protein (RFP) or its derivative mCherry (94) were constructed by in-frame integration at the corresponding chromosomal locus and expressed from the endogenous promoter. All such fusions were functional, as judged by the ability of the integrated construct to confer a normal phenotype. For routine visualization, yeast were grown to mid-exponential phase and viewed directly under an epifluorescence microscope (model BH-2; Olympus America, Inc., Center Valley, PA) using a 100× objective equipped with appropriate band-pass filters (Chroma Technology Corp., Rockingham, VT). Images were collected using a charge-coupled-device (CCD) camera (Photometrics, Inc., Tucson, AZ) and processed with μManager (95) and Photoshop (Adobe Systems, Inc., San Jose, CA). In some experiments, to demarcate the PM, cells grown to mid-exponential phase were stained with CellMask Orange (5 μg/ml; Thermo Fisher Scientific) for 2 min at room temperature, and then images were taken with an Elyra PS.1 structured illumination fluorescence microscope (Carl Zeiss, Jena, Germany) equipped with a Zeiss 100× PlanApo 1.46-numerical-aperture TIRF objective, a main focus drive of the AxioObserver Z1 Stand, a WSB PiezoDrive 08 for superresolution, and an Andor 512 × 512 EM-CCD camera (100-nm by 100-nm pixel size; Andor Technology, South Windsor, CT). GFP-tagged proteins were excited at 488 nm with an argon laser at 2.3% power (100 mW), and emission was monitored in a 495- to 550-nm window. CellMask Orange was excited at 561 nm at 2.3% power (100 mW), and emission was monitored in a 570- to 620-nm window. Images (average of 8 scans) were processed using ZEN software (Zeiss), ImageJ (NIH, Bethesda, MD) (96), and Photoshop (Adobe).

Actin patch dynamics was monitored essentially as described previously (97) at 25°C in a temperature-controlled environmental chamber (In Vivo Scientific, St. Louis, MO). Lifetimes were analyzed using ImageJ (NIH) and a plug-in that generates radial kymographs in 2-degree increments (http://www.embl.de/eamnet/html/body_kymograph.html).

## Supplementary Material

Supplemental material
