# Supplementary material for: TOR Complex 2-Regulated Protein Kinase Fpk1 Stimulates Endocytosis via Inhibition of Ark1/Prk1-Related Protein Kinase Akl1 in Saccharomyces cerevisiae
Source: Mol Cell Biol. 2017 Mar 17;37(7):e00627-16. doi: 10.1128/MCB.00627-16 (PMC5359421; doi:10.1128/MCB.00627-16)
Supplement: Supplemental material [file supp_37_7_e00627-16__index.html]

Supplemental material 

# TOR Complex 2-Regulated Protein Kinase Fpk1 Stimulates Endocytosis via Inhibition of Ark1/Prk1-Related Protein Kinase Akl1 in Saccharomyces cerevisiae

## Supplemental material

- Supplemental file 1 -

  Fig. S1 (Alk1 orthologs from 12 yeast species)

  PDF, 181K
- Supplemental file 2 -

  Movie S1 (Lifetime of Sla1-GFP at cortical actin patches)

  MOV, 1.2M
- Supplemental file 3 -

  Movie S2 (Lifetime of Sla1-GFP at cortical actin patches)

  MOV, 1.3M
