# Supplementary material for: TOR Complex 2-Regulated Protein Kinase Fpk1 Stimulates Endocytosis via Inhibition of Ark1/Prk1-Related Protein Kinase Akl1 in Saccharomyces cerevisiae
Source: Mol Cell Biol. 2017 Mar 17;37(7):e00627-16. doi: 10.1128/MCB.00627-16 (PMC5359421; doi:10.1128/MCB.00627-16)
Supplement: Supplemental material [file MCB.00627-16_zmb999101447s1.pdf]

Supplemental Figure 1

```
Sce  MSITNGTSRSVSAMGHPAVERYTPGHIVCVGTHKVEVVNYLAEGGFAQIYVVKFLEYLNEFDNTASV-PLKIGDVACLKRVLVQDENGLNEMRNEVEVMKKLKGAPNIVQYFD
Spa  MSITNGTSRSVSAMGHPAVERYTPGHIVCVGTHKVEVVNYLAEGGFAQIYVVKFLEYLNEFDNTASV-PLKIGDVACLKRVLVQDENGLNEMRNEVEVMKKLKGAPNVVQYFD
Sku  MSITNGTSRSVSAMGHPAVERYTPGDIVCVGTHKVEIVNYLAEGGFAQIYVVKFLEYLNEFDNAASV-PLKIGDLACLKRVLVHDENGLNEMRNEVEVMKKLKGAANVVQYYD
Smi  MSITNSTSRSVSAMGHPAVERFTPGHIVCVGTHKVEVINYLAEGGFAQIYMVKFVEYLNEFDNAASV-PLKIGDVACLKRVLVQDENGLNEMRNEVEVMKKLKGAPNVVQYFD
Sba  MSTTNGTSRSVSAMGHPAVERYTPGKIVCVGTHKVEIVNYLAEGGFAQIYAVKFLEYLNEFDNTASL-PLKIGDVACLKRVLVHDENGLNEMRNEVEVMKKLKGAANVVQYYD
Sar  MSITNGTSRSVSAMGHPAVERYTPGHIVCVGAHKVEVVNYLAEGGFAQIYVVKFLEYLNEFDNTASV-PLKIGDVACLKRVLVHDENGLNEMRNEVEVMKKLKGAANVVQYFD
Sca  MSTTSGPVSVNNTTVKPNNERYTPGTQVAVGAHKVEIIKYIAEGGFAQIYAVKFIEFLNEFENNRMKPKLQMGDVACLKRVLVQDENGLNEMRNEVEVMKQLQGAPNIVQYYD
Cgl  MERPN--SRSMTSLNSPNEEKYPNGQMISVGAHRVEIVSYLAEGGFAQIYVVKFVEYLNEFESLGSKSAITVGDIACLKRVIVNDEMGLNEMRNEVEVMKKLKSSPNIVQYFD
Ago  MSKRH(11)PGTGALTGPLEMLQAGSTVLVGVHQVEVIEYLAEGGFAHIYKVSFVGYTNELD-RQDR-ILQPGDTVCLKRVRVSDENGLNELRNEVEVMKKLRNCSNIVQYYD
Skl  MANSG(14)SASSSASPTLEKLAPGTLIIVGSHKVEIVKYLAEGGFAHIYVVKFVEFSNELE-TPSS-SLKEGDLACLKRVLVTDENGLNELRNEVEVMKQLKNSDNIVQYYD
Kwa  M-ITG(54)SSRSGTLPVLEQLKTGSQVIVGNHRVEIVKYLAEGGFAHIYVVRFIEYANELEQVPTI-KLEVGDLACLKRVLVTDENGLNEMRNEVSVMKQLSGCPNIVQYYD
Kla  MS(12)HTAATSAM-IPNSHLLSPNTQVVVGTHRCEILEHLAEGGFANIYKVKFLELTNEMDAGIDSKLLKAGDIACLKRVIVPDENGLNELRNEVETMKQLRGSPNIVQYYD

Sce  SNASRRRDGVQGFEVLLLMELCPNKSLLDYMNQRLSTKLTEAEIVKIMYDVALSISQMHYLPVSLIHRDIKIENVLVDAKNNFKLADFGSTSTCFPIVTTHQDIALLTQNIYV
Spa  SNASRRRDGVQGFEVLLLMELCPNKSLLDYMNQRLSTKLTESEIVKIMYDVALSISQMHYLPIPLIHRDIKIENVLVDAKNNFKLADFGSTSTCFPIVTTHQDIALLTQNIYV
Sku  SNASRRRDGVQGFEVLLLMELCPNKSLLDYMNQRLSTKLTETEIVKIMYDVALSISQMHYLPVPLIHRDIKIENVLVDAKDSFKLADFGSTSTCFPIVTTHQDIALLTQNIYV
Smi  SNASRRRDGVQGFEVLLLMELCPNKSLLDYMNQRLSTKLTESEIVKIMYDVVLSISQMHYLPVPLIHRDIKIENVLVDANNNFKLADFGSTSTCFPIVTTHQDIALLTQNIYV
Sba  SNASRRRDGVQGFEVLLLMELCPNKSLLDYMNQRLSTKLSESEIVKIMYDVALSISEMHYLPVPLIHRDIKIENVLVDAENNFKLADFGSTSTSFPIVTTHQDIALLTQNIYV
Sar  SNASRRRDGVQGFEVLLLMELCPNKSLLDYMNQRLSTKLTESEIVKIMYDVALSISEMHYLPVPLIHRDIKIENVLVDGENNFKLADFGSTSTCFPIVTTHQDIALLTQNIYV
Sca  SNASRRHNGFPGFEVLLLMELCPNKSLLDYMNQRLATKLTEKEILKIMYDVTYAVSQMHYLPTPLLHRDIKIENVLVDAQNNFKLCDFGSTSTKFPMVTTHQDIAVLTQNIYV
Cgl  SNASRRTDGKPGFEVLLLMELCPNKSLLDYMNQRLKTKLSESEILKIMYDVSIGISNMHYLDQPLIHRDIKIENVLVDANNNFKLCDMGSTSQCSPPMMSNQDIAMMTQNIYV
Ago  SNASRLGDGKPGYEVLLLMELCPNGSLLDYMNQRLATKLSEAEVLKIMYDITVGLSHMHYQRTPLIHRDIKIENVLVDADNNFKLCDFGSTSPCLPAVASHQEIAMLMNNIYV
Skl  SNASRRRDGSPGYEVLLLMELCPNKSLLDYMNQRLATKLSEKEVLKIMYDVTKAVAQMHFLPTPLIHRDIKIENVLVDSENNFKLCDFGSTSSCFPIMTTHQDIALLTNNIYV
Kwa  SHASRARDGSSGFEVTLLMELCPNNSLLDYMNQRLATKLSEQEILKIMFDVTRALAQMHYLPVPLIHRDVKIENVLVDANHNFKLCDFGSTSTCFPVANTHQEIAVLTNNIYV
Kla  SNASRHPDGSPGFEILLLMELCPKKSLLDYMNNKLATKLTEAEILKIMYDVSNAIAQMHYLPTPLIHRDIKIENVLVDKDDNFKLCDFGSTSMCFPIVSTHQDIAVLTNNIYV

Sce  HTTPQYRSPEMIDLYRCLPINEKSDIWALGVFLYKLLFFTTPFEMTGQFAILHSKYEFPVNKYSSKLINLIIIMLAENPNLRPNIYQVLYHLCEILNVEVPIEDKYAEGAYNF
Spa  HTTPQYRSPEMIDLYRCLPINEKSDIWALGIFLYKLLFFTTPFEMTGQFAILHSKYEFPVNKYSSKLINLIIIMLAENPNLRPNIYQVLYHLCEILNVEVPIEDKYTEGAYNF
Sku  HTTPQYRSPEMIDLYRCLPINEKSDIWALGIFLYKLLFFTTPFEMTGQFAILHSKYEFPVNKYSSKLINLIIIMLAENPNLRPNIYQVLCHLCDILNVEVPIEDKYAEGPYNF
Smi  HTTPQYRSPEMIDLYRCLPINEKSDIWALGIFLYKLLFFTTPFEMTGQFAILHSKYEFPVNKYSSKLINLIIIMLAENPNLRPNIYQVLYHLCEILNTEVPIEDKYTEGAYNF
Sba  HTTPQYRSPEMIDLYRCLPINEKSDIWALGIFLYKLLFFTTPFEMTGQFAILHSKYEFPVNKYSSKLINLIIIMLAENPNLRPNIYQVLYHICDILNAEVPIEDKYSEGAYDF
Sar  HTTPQYRSPEMIDLYRCLPINEKSDIWALGIFLYKLLFFTTPFEMTGQFAILHSKYEFPVNKYSSKLINLIIIMLAENPNLRPNIYQVLYHLCDILNVEVPIEDKYVEGAYNF
Sca  HTTPQYRSPEMIDLYRCLPIDEKSDIWALGIFLYKLLFFTTPFELTGQFAILHSKYEFPKNNYSSKLINLIIIMLAENPNLRPNIYQVLHNICSISGMKVPIEDQYAEGPYDF
Cgl  HTTPQYRAPEMIDLYRYLPINEKSDIWALGIFLYKLLFYTTPFEITGQMAILHSKYDFPPNKYSSKIINLIIIMLAENPNLRPNIFQVVYHICSIMNLPVPIEDRYGLGPYNF
Ago  HTTPQYRSPEMIDLYRCLPINEKSDIWALGIFLYKLLFYTTPFELTGQFAILHSKYEIPRNSFSSKLINLVIIMLAENPYLRPNVYQVMYHICSMMECEVKIDDLYGQGPYNF
Skl  HTTPQYRAPEMIDLYRCLPIDEKSDIWALGIFLYKLLFYTTPFELTGQFAILHSKYDIPANNYSSKLINLIIIMLAENPNLRPNIYQVIYQVCAIMKVDVPFEDKYQTGPYDF
Kwa  HTTPQYRSPEMIDLYRCLPINEKSDIWALGIFLYKLLFYTTPFELTGQFAILHSKYEFPVNNYSSKLINLIIIMLAENPNLRPNIYQVMDNLCSILKVKNPLEDKYQLGSYSF
Kla  HTTPQYRSPEMIDLYRCLPINEKSDIWALGIFLYKLLFYTTPFELTGQFAILHSKYEIPQNGYSSKLINLIIIMLAENPSLRPNIYQVMHHVCSILKCDVPFTDKYELGPYDF
```

```
Sce   SKYTQFQNKLQNVQLQMYQLQQKKIMQNNKLSDSEENLLNDMFLSSFEISSKLPMNAS-----DGHAAVSRIPSQNVGQELEEEKESQSDQRKSTLSEDKSSRTTSNANSSGT
Spa   SKYTQFQNKLQNVQLQMYQLQQKKIMQNNKLSDSEENLLNDMFLSSFEISSKLPMNVS-----DGPAAVSRISSQKVGKEVEEEKESHSDQRKSTLPEDKSSRLTSNVNSSGT
Sku   ARYTQFQNKLQNVQLQMYQLQQKKIVQNSKLSDSEENLLNDMFLSSFEISSKLPMNAS-----DGPMTVSRKASQNVNKEMEEKKESPSDQKKSAMSEEKASKIAPNIIDVGT
Smi   SKYTQFQNKLQNVQLQMYQLQQKKIMQNNKLSDSEENLLNDMFLSSFEISSKLPMNAS-----DGPVTVSKMASPNASKEAEEEKEGHFNQGESIVSEDKSSRVNSSVNNSSA
Sba   AKYTKFQNKLQNVQIQMYQLQQKKIMQNNKLSESEENLLNDMFLSSFEVSSKLPMNAS-----DGTVISGRAASQPIGKKTEEKEEPSEKRKSTVSEDKASRNTSNITDSSTG
Sar   GKYTQFQNKLQNVQLQMYQLQRKKIMQNNKLSNSEENLLNDMFLSSFEISSKLPMNAS-----DSSAVVSRTTSQNTGKEGEEKKEGTSEQRNPVTSEDKASRGTFSNTDSNT
Sca   EKYTHFQNKVQSVQYQLYLLQEKKFKTNGKLPQADINLLNDLFVTSFDIASKVPFELKVPTPLPGYSA--EIPESNFAKQEQQYEPRNIPTAGDYLNENRKSFTSNDALSRHT
Cgl   DLYTKFQSKVQTIQNQINYLQNKVVSSKSKLSKEDGLVLDELYIKTFEMIPKIPNPRL-----SEKQPTLEIGLNSPSIQDQHLDVNKN-KRR-SMHESINSRQASDNISQKS
Ago   DMYGRYQEKLQRLQYDMLMSHQ---LAQRGIINTDK--VNDLFISTFECAPKQPMVMGQNAVAQQQIFVAPPSTNTSMPVDMQQSLPPLDHNGPNAHGGL-------------
Skl   DKYSKYQEKLQAFQYQLYISYH-----QK--QDVDT--LNDLFINCFEIAPKQPMDMG-------KRDLAEKPENLEKIQNQQ------DSRKQI------------------
Kwa   EKYSQYQAKLQKVQYQMYLAYE------NK-QQIDT--LNDMFINCFEVAPKQPVDIS------------------------DKKW-ASGPSSYKTRENKTV-----------
Kla   AKYSQYHLKLQQIQYQMFELYK-----NEKVTSGDVDKLNDLFIQNFEIAPKQPIDKGIG---------------------------SVDQQE--------------------

Sce   ANNPQEINTIQSP--GIEDKSIFENKTPG------ELYYPSVSELDTYLDKELVKQSSDPTISEQSPRLNTQSLPQRQKSTSSYSSGGRSMKSTSYGAATIG---SDEALANE
Spa   PNNPQEINTIQSP--GIEDKSIFENKTPG------ELYYPSVSELDTYLDKELVKQSSDPTVSEQSPRLNTQSLPQRQKSTSSYSSGGRSMKSTSYGAATIG---SDEALTNE
Sku   SKDTQEINTVQSP--RIEDKSIFENKTPG------ELYYPSVSELDTYLDKELVKQPSDPTVSEKSPRLNTQSLPQRQKSTSSYSSGGRSMKSTSYGAATIG---SDEVLTNE
Smi   PNDLKEVNTIQSP--GIEDKSIFKNKTPG------ELYYPSVSELDTYLDKELVKQPSDPTVCEQSPRLNPQSLLQRQKSTSSYSSGGRSMKSTSYGAAAIE---SEEGLTNE
Sba   LNIPREINTIQSP--GIADKSIFEDKTPG------ELYYPSVSELDTYLDKEIVKQPSDPSVSDQNPHLNTQSAPQRQKSTSSYSSGGRSMKSTSYGAATIG---SEEALTNE
Sar   LNFPKEFSTIQSP--GIEDKSIFANKTPG------ELYYPSVSELDTYLDKELVKQPSDTTVSEQSPRLNAQSLPQRQKSTSSYSSGGRSMKSTSYGAATVG---SDEVLTNE
Sca   SNTADTSRDIATP-SNFEKKENVNEKSPGKPIENTEQYFPTVGELDYYLDNELKQQQQQQVHQ(46)LNTTGMVQRQKSLGSVSTDGRSVGSNSH-VTNAE---NLPTEYTA
Cgl   VRTS--SNTIEKTFSQTSNKEFSKETDPESHDESGEPYFPSVNELNTYLDKEFKQQKAQDNVVK(8)GNMNSSNPERQKSISSFSSSGKSMKSSSH-VPSAGFV-GADATEGP
Ago   ------------------------------------------DSLQKLPKSADVGNYP(15)HMQVPRKEVMMQHTDRSVLSDHSGNGTSTPSLPGSCPV--QH---EQLAN-
Skl   ---------------PIRDEEDI--KMV-------QQHFPSVEDLEIYLHSEA------THFENKSDRLSSLAVPNRSKSLGSSSHASSVTDLSNGSGNSPEEVSPGVMSSNP
Kwa   ---PEVNPDVTLA--GEEDIGLV------------QQQFPSVEDLDHYLDGEN-KRDTNKSSSNGAFSTEHSSGSSSKTDLSAIQHAPSVPLEELPSSKPTQNVQAV(7)KNM
Kla   ---------------APEDLETDIAKM--------DECYPTVEKLESANAKER-------------DDHHLQVLSTSLKQSSSRSISDLSNKSQV-SLDSNERHSSH(17)TA

Sce   KTAGINKMKQHKSNNPFPKMNVAYHSTNELSNDASNFFLEEQQQGQRYQQAQNQTGTQGNTFPDESQYQSRVEQQQQQQDQP--KGPANYSQRNFYTGRDRSN-KPMQLGGTI
Spa   KTPGINKMKQHKSNNPFPKMNVAYHSTNELSNDASNFFLEEQQQGQRYQQAQNQTGTQGNTFPDESQYQSRVEQHQQQQDQP--KAPANYNQRNFYTGRDRTN-KPMQLGGTV
Sku   KTAGINKMKQHKSNNPFPKMNVPYHSSNELSNDASNFFLEEQKQGQRYQQAQNQTGTQGNIYPEEAQYQSRVEQHQQQQDQS--QAPANYNQRAFYTGRDRATSKPMQLNGTV
Smi   KSVGINKMKQHKSNNPFPKMNVAYHSTNELNNDAGNFFLEEQQQSQRYQPAQNQTGAQGNIYPDESQYQSIVEQHQQQQDQP--KAPANYNQRNFYTGRDRAN-KPMQLGGTV
Sba   KNVGINKMKQHKSNNPFPKMNVAYHSTNELSNDASNFFLEEQQQNQRYQQAQNQPGAQGQSHPEEAQYQSRMEQ-QQQQDQP--QAPANYNQRAFYTGRDRAG-QSMQLGTTA
Sar   KTTGTSKMKQHKSNNPFPKINVAYQSTNELGKDANNFFLEEQQQNQRHQQTQNQPGAQGNAYSEEAQYQPRVEQQQHQQPQDQPQTPANYGQRGFYTGRDRAN-KSMQMGATV
Sca   KSDLPGMAKQHKSNNPFPKMTHAFQSANE---NVGTYFVDNNGSRQQVQQTE----------------------------------------ANMYTN----------LNT--
Cgl   KMVGYLKSQNHKSNNPFPFMAGDRQRSTESLNNKHNNYDANN--TTAPVK-GNYGGIQANNIPSQPK---NFEYNNPVNDTNRIQD-TGIGQSA---GRPQNVSQFSNTKMNF
Ago   -TP---KSKQYKKNNPFPKMAKQDFVH-DTYDESDEHSPGDD--PAPASK--PVDSMIPSVPATVTPMVSVQRDRSFQHIQP-GQIPENVRECEPESEVEMDLSHKIQNC-NL
Skl   TAKSTASIKQHKSNNPFPKM-------------------QQQQQPQQQQPQFYQQ--NIDYFHDTSKEAANPLALNAQQ------------IGYFHSIPTEAASKTQEIAYS-
Kwa   VRRSTNSVKQHKPTNPFPYIQ-PEKEIYGVDNDSNSIFHGEGSKSDPEQQYFAQDATA------VPQPPIKTEQPAQKQVGP--ALPTTEAAKSSL-KQFRA--QPQEPGG--
Kla   NMSAARQHKQHNPFQSQFQPEV----------DASEYFDANN------EQYFKGTP------------------------------PAHPKNE----KNVSYF----------
```

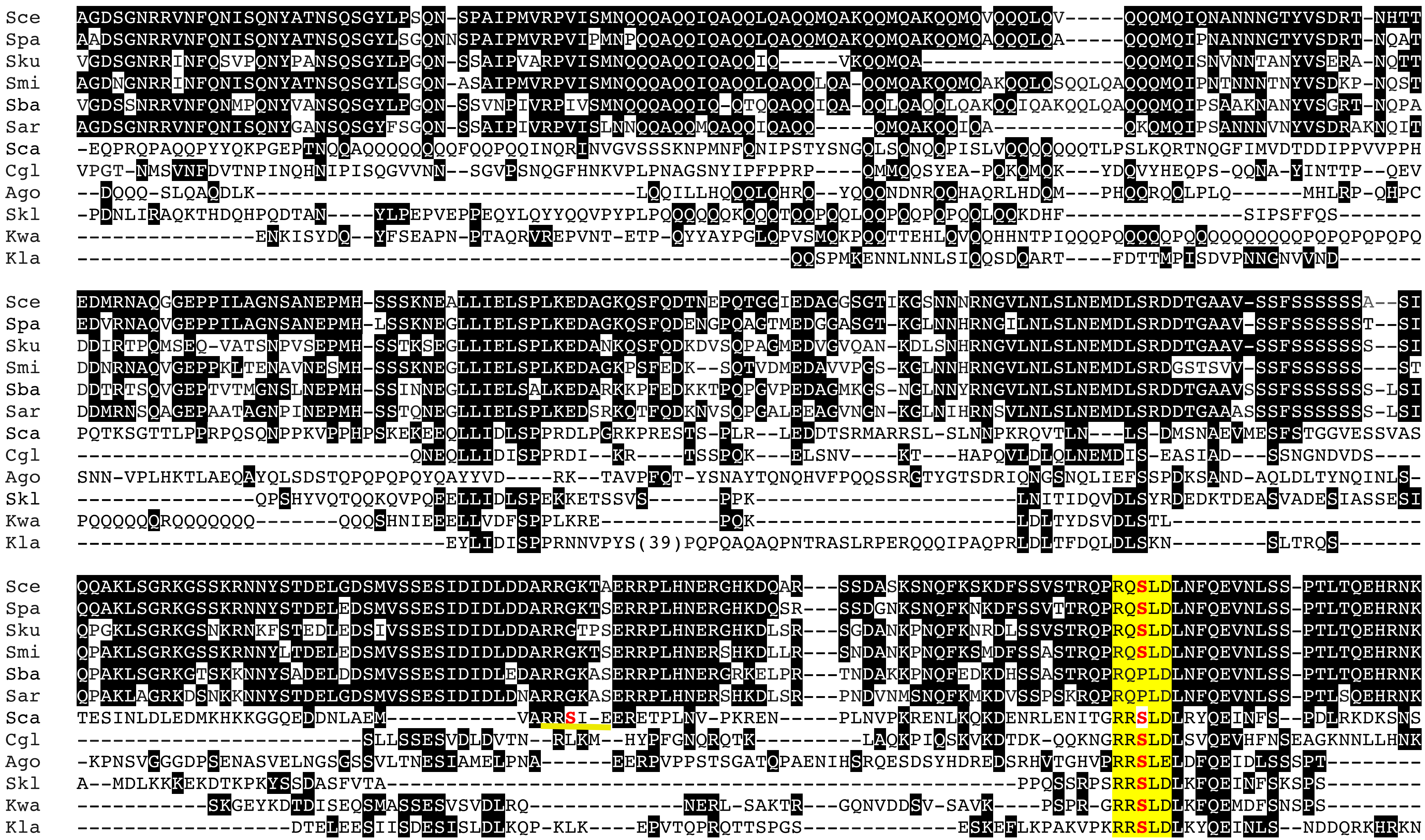
Sce AGDSGNRRVNFQNISQNYATNSQSGYLPSQN-SPAIPMVRPVISMNQQQAQQIQAQQLQAQQMQAKQQMQAKQQMQVQQQLQV-----QQQMQIQNANNNGTYVSDRT-NHTT
Spa AADSGNRRVNFQNISQNYATNSQSGYLSGQNNSPAIPMVRPVIPMNPQQAQQIQAQQLQAQQMQAKQQMQAKQQMQAQQQLQA-----QQQMQIPNANNNGTYVSDRT-NQAT
Sku VGDSGNRRINFQSVPQNYPANSQSGYLPGQN-SSAIPVARPVISMNQQQAQQIQAQQIQ-----VKQQMQA----------------QQQMQISNVNNTANYVSERA-NQTT
Smi AGDNGNRRINFQNISQNYATNSQSGYLSGQN-ASAIPMVRPVISMNQQQAQQIQAQQLQAQQLQA-QQMQAKQQMQAKQQLQSQQLQAQQQMQIPNTNNNTNYVSDKP-NQST
Sba VGDSSNRRVNFQNMPQNYVANSQSGYLPGQN-SSVNPIVRPIVSMNQQQAQQIQ-QTQQAQQIQA-QQLQAQQLQAKQQIQAKQQLQAQQQMQIPSAAKNANYVSGRT-NQPA
Sar AGDSGNRRVNFQNISQNYGANSQSGYFSGQN-SSAIPIVRPVISLNNQQAQQMQAQQIQAQQ-----QMQAKQQIQA-----------QKQMQIPSANNNVNYVSDRAKNQIT
Sca -EQPRQPAQQPYYQKPGEPTNQQAQQQQQQQFQQPQQINQRINVGVSSSKNPMNFQNIPSTYSNGQLSQNQQPISLVQQQQQQQTLPSLKQRTNQGFIMVDTDDIPPVVPPH
Cgl VPGT-NMSVNFDVTNPINQHNIPISQGVVNN--SGVPSNQGFHNKVPLPNAGSNYIPFPPRP----QMMQQSYEA-PQKQMQK---YDQVYHEQPS-QQNA-YINTTP--QEV
Ago --DQQQ-SLQAQDLK-------------------------------LQQILLHQQLQHRQ--YQQQNDNRQQHAQRLHDQM---PHQQRQQLPLQ------MHLRP-QHPC
Skl -PDNLIRAQKTHDQHPQDTAN----YLPEPVEPPEQYLQYYQQVPYPLPQQQQQQKQQQTQQPQQLQQPQQPQPQQLQQKDHF---------------SIPSFFQS-------
Kwa ----------------ENKISYDQ--YFSEAPN-PTAQRVREPVNT-ETP-QYYAYPGLQPVSMQKPQQTTEHLQVQQHHNTPIQQQPQQQQQPQQQQQQQQQPQPQPQPQPQ
Kla ---QQSPMKENNLNNLSIQQSDQART----FDTTMPISDVPNNGNVVND-------

Sce EDMRNAQGGEPPILAGNSANEPMH-SSSKNEALLIELSPLKEDAGKQSFQDTNEPQTGGIEDAGGSGTIKGSNNNRNGVLNLSLNEMDLSRDDTGAAV-SSFSSSSSSA--SI
Spa EDVRNAQVGEPPILAGNSANEPMH-LSSKNEGLLIELSPLKEDAGKQSFQDENGPQAGTMEDGGASGT-KGLNNHRNGILNLSLNEMDLSRDDTGAAV-SSFSSSSSST--SI
Sku DDIRTPQMSEQ-VATSNPVSEPMH-SSTKSEGLLIELSPLKEDANKQSFQDKDVSQPAGMEDVGVQAN-KDLSNHRNGVLNLSLNEMDLSRDDTGAAV-SSFSSSSSSS--SI
Smi DDNRNAQVGEPPKLTENAVNESMH-SSSKNEGLLIELSPLKEDAGKPSFEDK--SQTVDMEDAVVPGS-KGLNNHRNGVLNLSLNEMDLSRDGSTSVV-SSFSSSSSS---ST
Sba DDTRTSQVGEPTVTMGNSLNEPMH-SSINNEGLLIELSALKEDARKKPFEDKKTPQPGVPEDAGMKGS-NGLNNYRNGVLNLSLNEMDLSRDDTGAAVSSSFSSSSSSS-LSI
Sar DDMRNSQAGEPAATAGNPINEPMH-SSTQNEGLLIELSPLKEDSRKQTFQDKNVSQPGALEEAGVNGN-KGLNIHRNSVLNLSLNEMDLSRDDTGAAASSSFSSSSSSS-LSI
Sca PQTKSGTTLPPRPQSQNPPKVPPHPSKEKEEQLLIDLSPPRDLPGRKPRESTS-PLR--LEDDTSRMARRSL-SLNNPKRQVTLN---LS-DMSNAEVMESFSTGGVESSVAS
Cgl ------------------------------QNEQLLIDISPPRDI--KR----TSSPQK---ELSNV----KT---HAPQVLDLQLNEMDIS-EASIAD---SSNGNDVDS----
Ago SNN-VPLHKTLAEQAYQLSDSTQPQPQPQYQAYYVD----RK--TAVPFQT-YSNAYTQNQHVFPQQSSRGTYGTSDRIQNGSNQLIEFSSPDKSAND-AQLDLTYNQINLS-
Skl ---------------QPSHYVQTQQKQVPQEELLIDLSPEKKETSSVS------PPK----------------------LNITIDQVDLSYRDEDKTDEASVADESIASSESI
Kwa PQQQQQQRQQQQQQQ-------QQQSHNIEEELLVDFSPPLKRE-----------PQK----------------------LDLTYDSVDLSTL----------------------
Kla ---------------------------------EYLIDISPPRNNVPYS(39)PQPQAQAQPNTRASLRPERQQQIPAQPRLDLTFDQLDLSKN---------SLTRQS-------

Sce QQAKLSGRKGSSKRNNYSTDELGDSMVSSESIDIDLDDARRGKTAERRPLHNERGHKDQAR---SSDASKSNQFKSKDFSSVSTRQPRQSLDLNFQEVNLSS-PTLTQEHRNK
Spa QQAKLSGRKGSSKRNNYSTDELEDSMVSSESIDIDLDDARRGKTSERRPLHNERGHKDQSR---SSDGNKSNQFKNKDFSSVTTRQPRQSLDLNFQEVNLSS-PTLTQEHRNK
Sku QPGKLSGRKGSNKRNKFSTEDLEDSIVSSESIDIDLDDARRGTPSERRPLHNERGHKDLSR---SGDANKPNQFKNRDLSSVSTRQPRQSLDLNFQEVNLSS-PTLTQEHRNK
Smi QPAKLSGRKGSSKRNNYLTDELEDSMVSSESIDIDLDDARRGKTSERRPLHNERSHKDLLR---SNDANKPNQFKSMDFSSASTRQPRQSLDLNFQEVNLSS-PTLTQEHRNK
Sba QPAKLSGRKGTSKKNNYSADELDDSMVSSESIDIDLEDARRGKASERRPLHNERGRKELPR---TNDAKKPNQFEDKDHSSASTRQPRQPLDLNFQEVNLSS-PTLTQEHRNK
Sar QPAKLAGRKDSNKKNNYSTDELGDSMVSSESIDIDLDNARRGKASERRPLHNERSHKDLSR---PNDVNMSNQFKMKDVSSPSKRQPRQPLDLNFQEVNLSS-PTLSQEHRNK
Sca TESINLDLEDMKHKKGGQEDDNLAEM------------VARRSI-EERETPLNV-PKREN-----PLNVPKRENLKQKDENRLENITGRRSLDLRYQEINFS--PDLRKDKSNS
Cgl ---------------------------SLLSSESVDLDVTN--RLKM--HYPFGNQRQTK---------LAQKPIQSKVKDTDK-QQKNGRRSLDLSVQEVHFNSEAGKNNLLHNK
Ago -KPNSVGGGDPSENASVELNGSGSSVLTNESIAMELPNA------EERPVPPSTSGATQPAENIHSRQESDSYHDREDSRHVTGHVPRRSLELDFQEIDLSSSPT--------
Skl A--MDLKKKEKDTKPKYSSDASFVTA---PPQSSRPSRRSLDLKFQEINFSKSPS---------
Kwa -----------SKGEYKDTDISEQSMASSESVSVDLRQ--------------NERL-SAKTR---GQNVDDSV-SAVK----PSPR-GRRSLDLKFQEMDFSNSPS---------
Kla ------------------DTELEESIISDESISLDLKQP-KLK----EPVTQPRQTTSPGS-------------ESKEFLKPAKVPKRRSLDLKYQEINLS--NDDQRKHRKN

```
Sce   NDSPAPNSHHSYRVSPHASTAITENKRHSTGHELSTRSNGKHETHRTGSKQRHDLERYRHSKDKDSNSSITISTSTPSEMRKSFARARQSLDLERVRREAMAS--SASSSGGS
Spa   NDSSAPQPHHSYRVPPHASTAISENKRHSTGHELSTRSSGKHETHRTGSKQRHDLERYRQSKDKDSNSSITISTSTPSEMRKSFARARQSLDLERVRREAMAS--NGSNSGGS
Sku   NDSSAPQSHHSHRVPPHTSTAISENKRHSTGHDSSTRLSAKHEAHRTSSKPRHDLERYRQSKDRDSNSSITISTSTPSEMRKSFARARQSLDLERVRRETMAS--NGTSSGGS
Smi   NESLAPQSHHSYRAPSHASTAISENKRHSTGHELSTRSTGKQDTHRTSSKQRHDLERYRHSKDKDSNSSITISTSNSSEMRKSFARARQSLDLERVRREAMAS--NGSSSGSS
Sba   TGSPAPQSHHSHRVPPHSSTTISENKRHSTGHE-STRSSGKHEMHRTNSKPRHDLERYRQSKDRDSNSSITISTSTPSEMRKSFARARQSLDLERIRREATANNGS-----GS
Sar   NDSQASQSHHSHRGPPHSSTTMTENKRHSTGHE-STRSSGKHETHRTNSKPRHDLERYRKSKDRDSNSSITISTSTPSEMRKSFARARQSLDLERVRREAMASSG--SGSGSS
Sca   STSVHHISSIRE(19)QRQQPRHHQHTHFDSSSNLNSKGANRSSSRGNIRGKQDLESYKHSTKNNSNSSIPISTTNTNEMKKSFAKARQSLDLERVRREALL--------NSD
Cgl   NTHRPGIKGSMS(20)QSSKVISQSTNARTSLDRQRQRHIEPRDSKSRSRS-HGLEEY-TSSNG-SNSSINISTSNKFEMKRSFAKARQSLDLERARRDAM----SRSNSGHE
Ago   ---------------------------------------PVSASKTSSKAHLQPNRSGTANCGTSN------SSSVVSGVRKSFHRGRKSVDLDVSKKE---SKEEPTNSG--
Skl   -----------------------------------PKESSQRHQHKESSQRHQHKKI---------------PSS--SEMRKSLSRARKSLDLEGVKRE------SAGGSD-T
Kwa   ------------------------------------PDIKLQKTKTVSHQQR--------------------KSNVAAEPKKSFSRARKSLDLERTKRD------TPSNGENT
Kla   ------------------------------------------------------------------------KTSF------SRTSVRRSVEMERMKHDSNSTSNSNARDETK

Sce   NGKRRSFFSVFRSEK.
Spa   NGKRRSFFSVFRSEK.
Sku   SGKRRSFFSVFRSEK.
Smi   NGKRRSFFSVFRSEK.
Sba   SGKRRSIFSVFRSEK.
Sar   GGKRRSIFSVFRSEK.
Sca   NGKRRSIFSMFRGDKK.
Cgl   TGKRKSLFSMFK.
Ago   SGKRRSIFGVFKS.
Skl   TSKRKSFFGVFKS.
Kwa   SSKRKSFFGKFKG.
Kla   ETKRRSFFGVFK.
```

Figure S1. Comparison of Alk1 orthologs from twelve yeast species. The amino acid sequence of *Saccharomyces cerevisiae* Akl1 (*top line*) was aligned with the primary structures of the Alk1 orthologs from eleven other yeast species, including the *sensu stricto* group, *Saccharomyces paradoxus* (Spa), *Saccharomyces kudriavzevii* (Sku), *Saccharomyces mikatae* (Smi), *Saccharomyces bayanus* (Sba), and *Saccharomyces arboricola* (Sar), as well as more divergent species, *Saccharomyces castellii* (Sca), *Candida glabrata* (Cgl), *Ashbya gossypii* (Ago), *Saccharomyces kluyveri* (Skl), *Kluveromyces waltii* (Kwa), and *Kluveromyces lactis* (Kla). As a means to emphasize their degree of relatedness to *S. cerevisiae* Akl1, only identities between the indicated ortholog and *S. cerevisiae* Akl1 are indicated (*white letters on black boxes*). Gaps (*hyphens*), as well as the positions of insertions of the indicated length (*in parentheses*), introduced to maximize the alignment in certain regions are also indicated. Period (.) indicates the end of the open-reading-frame. Matches to the consensus Fpk1 phospho-acceptor site motif (-R-x-S-L/V/I-D/E-) (*yellow boxes with phosphorylation site in bold red*); additional site in, respectively, Sca and Clg (*yellow underline*). The sources of the sequences shown were: Sce, strain S288C from the Saccharomyces Genome Datbase (http://www.yeastgenome.org/locus/S000000263/protein); Spa, strain CBS432 derived from the data of Liti *et al.* (2009) and Bergström *et al.* (2014); Sku, Smi, Sba, Sca and Skl as reported by Cliften *et al.* (2003) and Kellis *et al.* (2003); Sar from GenBank entry EJS44833.1; and, Cgl, Ago, Kwa and Kla from the Fungal Orthogroups database at the Broad Institute (https://portals.broadinstitute.org/cgi-bin/regev/orthogroups/show_orthogroup.cgi?orf=YBR059C).

SUPPLEMENTAL REFERENCES

Bergström A, Simpson JT, Salinas F, Barré B, Parts L, Zia A, Nguyen Ba AN, Moses AM, Louis EJ, Mustonen V, Warringer J, Durbin R, Liti G (2014) A high-definition view of functional genetic variation from natural yeast genomes. *Mol. Biol. Evol*. **31**: 872-888.

Cliften P, Sudarsanam P, Desikan A, Fulton L, Fulton B, Majors J, Waterston R, Cohen BA, Johnston M (2003) Finding functional features in Saccharomyces genomes by phylogenetic footprinting. *Science* **301**: 71-76.

Kellis M, Patterson N, Endrizzi M, Birren B, Lander ES (2003) Sequencing and comparison of yeast species to identify genes and regulatory elements. *Nature* **423**: 241-254.

Liti G, Carter DM, Moses AM, Warringer J, Parts L, James SA, Davey RP, Roberts IN, Burt A, Koufopanou V, Tsai IJ, Bergman CM, Bensasson D, O'Kelly MJ, van Oudenaarden A, Barton DB, Bailes E, Nguyen AN, Jones M, Quail MA, Goodhead I, Sims S, Smith F, Blomberg A, Durbin R, Louis EJ (2009) Population genomics of domestic and wild yeasts. *Nature* **458**: 337-341.
